# Multi-Source Imaging and Machine Learning for Non-Destructive Quality Assessment of Edible Root and Tuber Crops: A Review

**DOI:** 10.3390/foods15213903

**Published:** 2026-10-30

**Authors:** Emerson Asto-Rodriguez, Gian Pierre Silvera-Otañe, César Samaniego-Rafaele, Patrick A. Obregón, Guillermo Kemper

**Affiliations:** 1 Programa de Doctorado en Ingeniería Agroindustrial, Mención Transformación Avanzada de Granos y Tubérculos Andinos, Universidad Nacional del Santa, Nuevo Chimbote 02712, Peru; 2025818021@uns.edu.pe (E.A.-R.); 2025818011@uns.edu.pe (G.P.S.-O.); 2025818007@uns.edu.pe (C.S.-R.); 2025818004@uns.edu.pe (P.A.O.); 2 ARCAM-UNT Research Group, Universidad Nacional de Trujillo, Av. Juan Pablo II s/n, Trujillo 13011, Peru; 3 Faculty of Engineering, School of Electronic Engineering, Universidad Peruana de Ciencias Aplicadas (UPC), Av. Prolongación Primavera 2390, Monterrico, Santiago de Surco, Lima 15023, Peru

**Keywords:** edible tubers, potato, deep learning, hyperspectral imaging, object detection, non-destructive quality assessment, postharvest grading

## Abstract

Edible roots and tubers, such as potato, sweetpotato, cassava, and carrot, are vital to global food security; however, postharvest quality control still relies largely on costly, subjective manual inspection. Computer vision and machine learning applied to multiple imaging modalities have emerged as non-destructive approaches for automated inspection and quality assessment. This review synthesizes 141 studies (2016–2026) that applied these methods to the non-destructive quality assessment of edible roots and tubers. The reviewed studies span eight species, with potato (77.3%) and sweetpotato (17.0%) being dominant. RGB imaging is the most common acquisition modality (79%), alongside the growing use of hyperspectral and near-infrared spectroscopy. Deep learning is now the default approach, with its share rising from 18% (2016–2020) to 71% (2024–2026). YOLO-family detectors lead real-time sorting and defect detection, while classical image processing and machine learning remain competitive for narrowly defined tasks such as size grading. Several gaps stand out. Evidence beyond potato and sweetpotato remains thin. Public benchmarks are almost nonexistent, with only 5 of 141 studies using a public dataset. Foundation and zero-/few-shot models were almost absent from the reviewed studies, and most studies measure benchtop accuracy rather than the full inspection-to-actuation pipeline. This review maps where the field has matured and where it has not, offering a structured reference point and a research agenda for non-destructive quality evaluation of these crops.

## 1. Introduction

Edible roots and tubers, including potato (*Solanum tuberosum* L.), sweetpotato (*Ipomoea batatas* L.), cassava (*Manihot esculenta* Crantz), and carrot (*Daucus carota*), play an important role in global food security and the sustainability of agricultural systems. Potato ranks as the fourth most important food crop worldwide after wheat, rice, and maize [1], constituting a resource of high nutritional value owing to its richness in starch, protein, potassium, calcium, and vitamin C [2]. In turn, sweetpotato ranks seventh in agricultural production volume worldwide, contributing complex carbohydrates, dietary fiber, minerals, and β-carotene. China leads the global cultivation of this crop, producing approximately 80% of the world’s supply [3]. Cassava, widely grown across tropical regions of Latin America, Asia, and sub-Saharan Africa, is the main caloric source for more than 800 million people worldwide owing to its high starch content, with Thailand standing out as the second-largest producer and the world’s leading exporter [4]. Finally, carrots are among the most widely cultivated root vegetables, valued for their nutritional content, particularly fiber, essential nutrients, and β-carotene [5]. Together, these four crops substantially contribute to global caloric and nutritional intake, particularly in regions where food security remains a concern.

Despite their agronomic and socioeconomic importance, the supply and postharvest chain of these crops faces challenges that affect their commercial viability. During growth and harvest, these roots and tubers are vulnerable to pests, insect infestation, and mechanical damage that degrade their external quality, resulting in economic losses [3]. In potato, problems such as sprouting during storage render the product unusable for the processing industry [6], while skinning injury not only causes discoloration but also opens pathways for pathogenic infection and increases water loss during storage [7]. The onset of postharvest physiological deterioration limits the shelf life of cassava, ranging from optimal freshness to advanced degradation characterized by cracks and dark spots [4]. Carrot growth anomalies generate geometric variability, natural curvature, and irregularities that complicate processing and classification [5]. These postharvest defects are largely visual, which is precisely what makes them, in principle, detectable through image-based inspection.

Traditionally, the quality control, sorting, and postharvest classification of these roots and tubers have relied on manual visual inspection by human operators [8]. This manual approach has several drawbacks. It is costly, accounting for up to 50% of the total labor costs in packing lines [9]. It is also time-consuming, subjective, and prone to inconsistency due to worker fatigue and stress. The repetitive nature of these tasks also poses occupational health risks, causing physical discomfort and musculoskeletal disorders [10]. Similarly, traditional mechanical sorting systems based solely on sieve or hole size often fail to classify pieces with complex deformations or natural geometric irregularities [5]. These constraints on manual and mechanical grading motivate the search for automated, consistent, and non-contact inspection methods.

Computer vision (CV) and machine learning (ML) have emerged as non-destructive technologies for automating and standardizing industrial classification [3,11]. Throughout this review, CV denotes image-based acquisition and analysis across different sensor types, including red-green-blue (RGB), hyperspectral, multispectral, depth, and thermal imaging. These sensors can be used alone or combined to capture complementary information from the same tuber; we refer to this range of modalities as multi-source imaging. RGB cameras record surface color, shape, and texture; hyperspectral and multispectral cameras record reflectance across narrow bands, allowing internal composition such as starch or moisture to be inferred [12,13]. Depth cameras add geometry and volume [10,14]; thermal cameras reveal bruising and storage-related changes [4,15]; laser backscattering and biospeckle imaging characterize tissue using coherent light [7,16]; and computed tomography and magnetic resonance imaging resolve internal defects [17]. Some systems combine several sources, such as RGB with depth [18] or with hyperspectral imaging [19], while others add non-imaging sensors, typically near-infrared (NIR) point spectrometers [20,21,22]. Because each sensor captures distinct attributes, its choice shapes which inspection tasks and learning algorithms are feasible.

Early approaches in this field relied mainly on classical digital image processing, typically involving segmentation of the tuber from its background through color-space analysis and thresholding, followed by morphological operations to remove background noise and refine object contours [14,23]. These classical pipelines can achieve good results under controlled acquisition conditions, but manual parameter tuning is generally required for each new crop, camera, or lighting setup. Once a clean binary representation of the tuber was obtained, these classical systems extracted handcrafted geometric and texture descriptors, which were then coupled with supervised machine learning classifiers such as Support Vector Machines, Random Forest, or Artificial Neural Networks to perform tasks such as variety classification, quality grading, or deterioration assessment [24,25]. Although effective for narrowly defined, low-variability inspection tasks, this reliance on manually engineered features limited the generalizability of classical pipelines to the wide morphological and chromatic diversity naturally present in root and tuber crops.

In recent years, the introduction of deep learning (DL) has reshaped the field by enabling the automatic extraction of complex, hierarchical features directly from RGB (Red-Green-Blue) or multispectral images, thereby overcoming the limitations of classical methods in terms of generalization [26]. Convolutional neural network (CNN) models, such as VGG, ResNet, DenseNet, Xception, and Inception, have been implemented with high accuracy to identify storage pathologies in potato tubers [27]. Likewise, real-time object detection and instance-segmentation algorithms have enabled the automation of continuous transport lines. In particular, the You Only Look Once (YOLO) architecture is used to track and estimate the physical dimensions and defects of multiple moving samples [10], while Mask R-CNN, a region-based convolutional neural network (R-CNN) variant, can segment irregularly shaped tubers against complex, low-contrast backgrounds, such as field soil, during mechanized harvest [28]. Collectively, these deep learning-based approaches have been proposed to narrow the gap between the performance achieved under controlled laboratory conditions and the variability encountered on real industrial packing lines.

### 1.1. Related Reviews and the Gap This Review Addresses

Several previous reviews cover parts of this topic. They are not part of the 141 primary studies analyzed here and are cited as context. Imaging and spectroscopy for potato and sweetpotato quality have been reviewed [29,30], and potato has been addressed through imaging and artificial intelligence for quality classification [31], deep learning along the production chain [32], hyperspectral imaging [33], and machine vision from cultivation to storage [34]. For other root and tuber crops, the available reviews address one or two crops each, namely cassava and yam with near-infrared hyperspectral imaging [35] and sweetpotato with spectroscopy and imaging coupled with machine learning [36]. Five of the eight focus on the harvested organ, and the remaining three also cover plants in the field. Table 1 summarizes their scope.

These reviews address one or two crops each, and several cover the whole production chain or a single sensing family. We found no review that maps the evidence on the harvested organ across root and tuber crops and across imaging modalities. This review adds three elements. First, it centers on the harvested organ and, with a search built on generic root and tuber terms in addition to the main crop names, maps how the studies are distributed across crops. Second, it brings together RGB, spectral, and depth imaging with technique families ranging from classical image processing to deep learning, matched to the inspection task, and follows how the technique mix has changed over ten years. Third, it describes how the reviewed systems are set up for real use: the acquisition environment (laboratory, field, or packing line), the number of views captured per tuber, imaging of moving samples on conveyors, the inference hardware (for example, GPU or embedded devices), and integration with programmable logic controllers (PLC) or actuators that sort or reject tubers.

### 1.2. Scope of This Review

This review covers literature published from 2016 to 2026, a decade of research, synthesizing computational systems based on digital image processing, traditional machine learning, and deep learning applied to multi-source image data (RGB, hyperspectral, multispectral, depth, thermal, and other modalities) for the postharvest quality assessment of edible roots and tubers. It brings together studies on potato, sweetpotato, cassava, carrot, and other minor root and tuber crops within a single analytical framework. Over this period, these techniques have been progressively extended to an increasing number of crops and inspection tasks, with continuous improvements in accuracy, speed, and robustness. Through this analysis, we examine the operational advantages reported for each methodological family, the most frequently used software architectures, and the challenges that remain toward their practical implementation in packing lines.

The remainder of this review is organized as follows. Section 2 describes the literature search, study selection, and data extraction, and Section 3 gives an overview of the included studies. Section 4 covers the data acquisition systems, Section 5 the classical image processing techniques, and Section 6 machine learning and deep learning. Section 7 and Section 8 present the applications by inspection task and by crop, respectively. Section 9 examines datasets and evaluation metrics, Section 10 discusses trends, challenges, and limitations, Section 11 proposes future research directions, and Section 12 presents the conclusions.

## 2. Materials and Methods

This study is a narrative review based on a structured literature search. No protocol was prepared or registered. The structured search was used to make the identification and selection of studies transparent and reproducible. The studies were synthesized thematically, not quantitatively. They were organized according to acquisition modality, technique family, and inspection task, with no formal meta-analysis.

Two bibliographic databases, Scopus and Web of Science (Core Collection), were searched on 17 July 2026. Both were chosen because they provide consistent metadata and a more reproducible search. Only journal articles were included. Each search combined three concept blocks using Boolean operators. The first covered computer vision and machine learning, the second covered the major edible root and tuber crops along with generic tuber and root terms, and the third covered postharvest tasks. The complete search equation was as follows:

*(*“*computer vision*” *OR* “*machine vision*” *OR* “*deep learning*” *OR* “*convolutional neural network*” *OR* “*image processing*” *OR* “*image analysis*” *OR* “*hyperspectral imaging*” *OR* “*machine learning*”*) AND (*“*potato*” *OR* “*potatoes*” *OR* “*cassava*” *OR* “*manihot esculenta*” *OR* “*sweet potato*” *OR* “*sweet potatoes*” *OR* “*ipomoea batatas*” *OR* “*yam*” *OR* “*yams*” *OR* “*dioscorea*” *OR* “*tuber*” *OR* “*tubers*” *OR* “*tuberous root*” *OR* “*tuberous roots*” *OR* “*storage root*” *OR* “*storage roots*” *OR* “*root crop*” *OR* “*root crops*” *OR* “*root vegetable*” *OR* “*root vegetables*”*) AND (*“*quality assessment*” *OR* “*quality evaluation*” *OR* “*quality inspection*” *OR* “*grading*” *OR* “*sorting*” *OR* “*defect detection*” *OR* “*disease detection*” *OR* “*classification*” *OR* “*postharvest*” *OR* “*post-harvest*” *OR* “*harvesting*”*)*

In Scopus, the query was applied to the title, abstract, and keywords (TITLE-ABS-KEY), restricted to the document-type article, with the publication-year filter PUBYEAR >2014 AND PUBYEAR <2027 (years 2015 to 2026). The equivalent topic-field query in the Web of Science was restricted to the article document type, with a publication-date filter from 1 January 2015 to 1 January 2027. Both date filters were set intentionally wider than the substantive coverage window of the review, as a precaution against missing any eligible article indexed close to the search date; the single official search date for both databases was 17 July 2026.

Studies were eligible if they used computer vision or image-based sensing to assess the root and tuber external appearance, internal quality, defects, diseases, or physiological traits. Measurements had to be non-destructive and applied directly to the organ itself. Studies analyzing leaves, stems, or other plant parts were retained only when the tuber or root was also evaluated. No language restrictions were applied. Studies that focused exclusively on leaves, stems, other plant parts, whole plants, or field plots, used destructive assays, or were reviews, conference abstracts, or theses were excluded. The previous reviews presented in Table 1 were not part of this corpus; they were identified in a separate search using the same equation, restricted to the review document type in Scopus and Web of Science, on 16 October 2026, and are cited in the Introduction and the Discussion as context.

**Figure 1:**
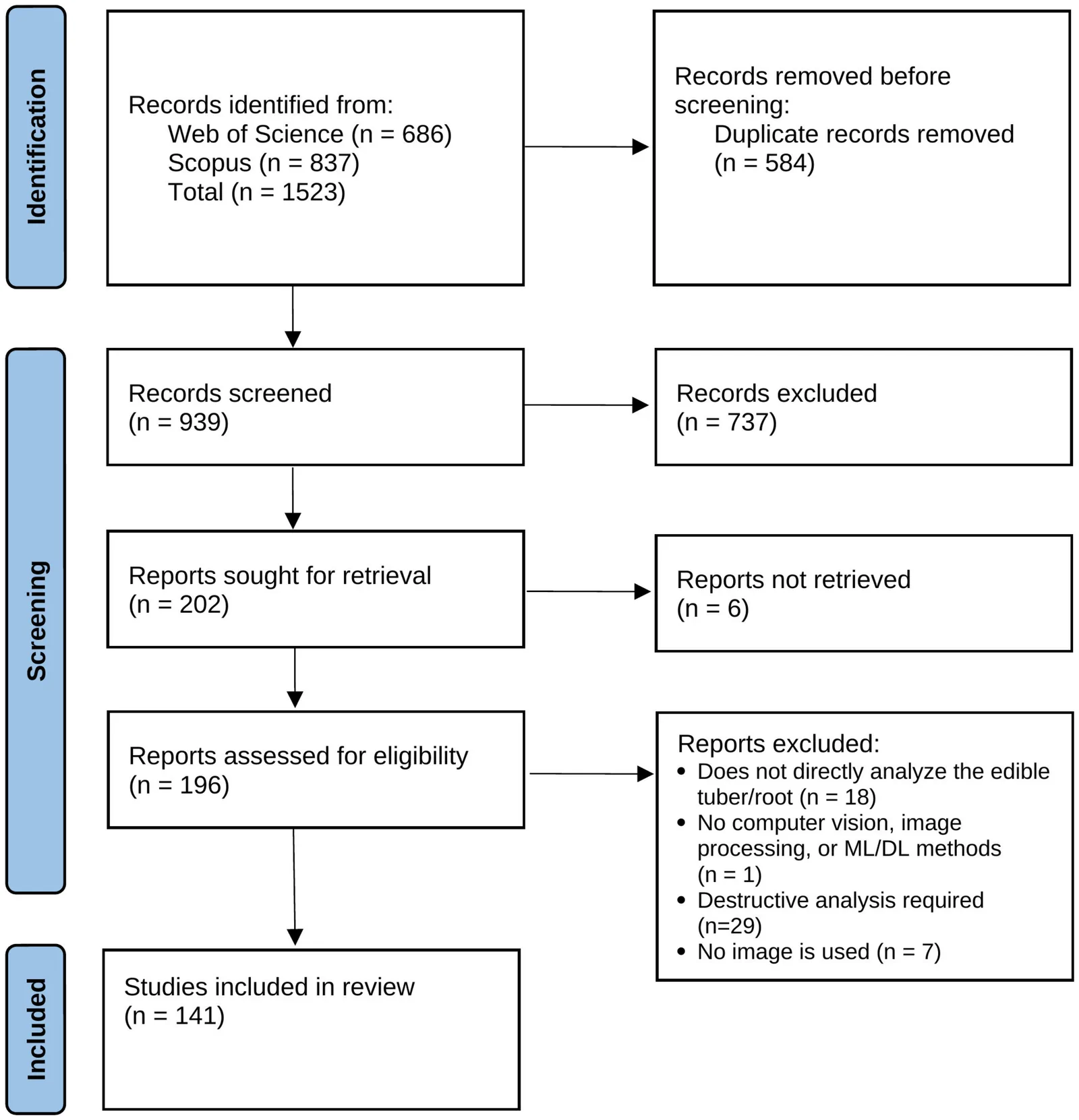
Flow diagram of the literature search and study selection, showing identification, screening, eligibility, and inclusion.

Figure 1 summarizes the identification, screening, and eligibility process through a flow diagram of the literature search and selection. Of the 1523 records identified across the two databases, 141 studies remained after duplicate removal, successive rounds of title and abstract screening, and full-text eligibility assessment. Duplicate removal was performed algorithmically based on the DOI or on title similarity when the DOI was not available. Most full-text exclusions occurred because the analysis required physical destruction of the organ or because the study examined only non-tuber plant parts.

For each included study, a structured set of variables was extracted and coded, covering bibliographic data, the study’s stated aim, the crop and organ studied, the image- or spectroscopy-based acquisition setup, the image-processing or modeling pipeline, the evaluation metrics reported, any comparison against classical, non-deep-learning methods, and the study’s own reported findings and limitations. Additional variables included the acquisition environment, the number of views per tuber, and whether imaging was performed during movement, such as on conveyors, rollers, or harvesters. Hardware information was also extracted when reported, including the processing platforms used for model development and inference, edge or embedded devices, and connections with industrial control or actuation systems. This coded dataset underlies the counts and cross-tabulations reported in this review.

A descriptive bibliometric analysis was also conducted to characterize research trends among the included studies. This analysis examined publication trends, the geographic distribution of studies based on corresponding authors’ affiliations, the crop species investigated, and author keywords. Keyword co-occurrence was analyzed using VOSviewer (version 1.6.21) to identify research themes and examine their evolution over time. Singular, plural, and synonymous terms were standardized using a thesaurus available in the Zenodo repository, and a minimum threshold of four occurrences was applied. The bibliometric analysis provides an overview of the literature before the detailed synthesis in Section 4, Section 5, Section 6, Section 7, Section 8, Section 9, Section 10 and Section 11 and was not used to determine study inclusion or assign weights to individual studies.

## 3. Overview of the Selected Studies

### 3.1. Publication Trends and Geographic Origins

**Figure 2:**
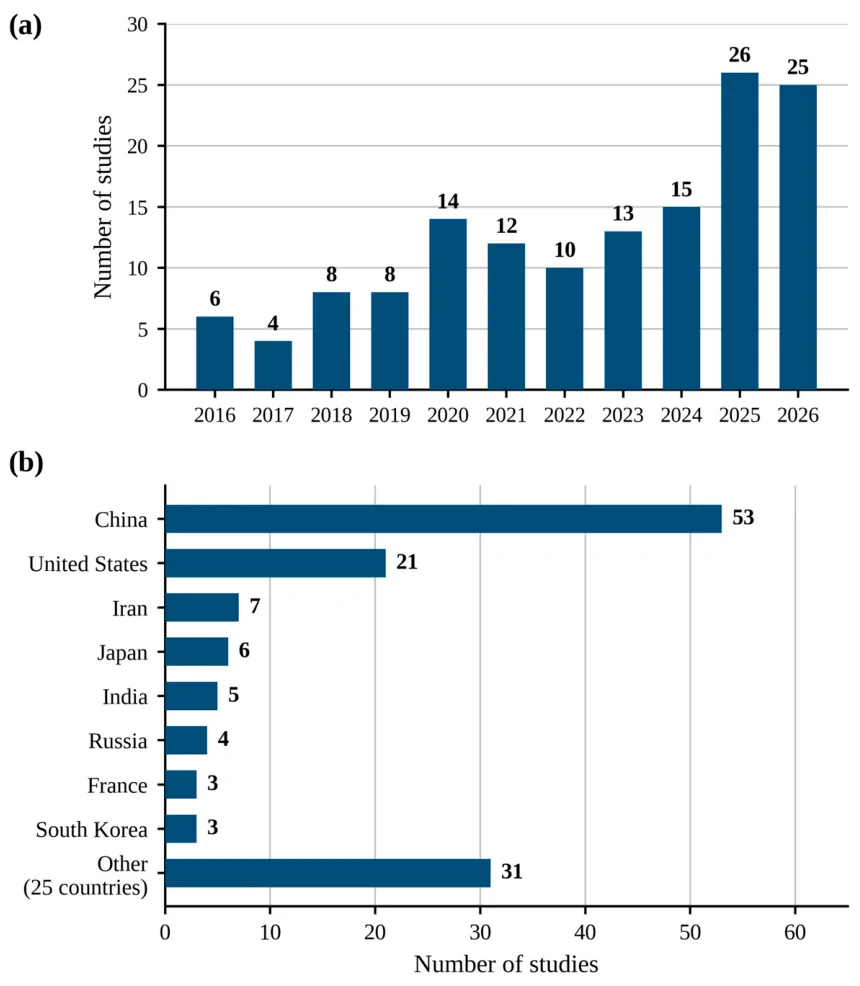
(**a**) Number of studies included per year and (**b**) number of studies included per corresponding author country/region, 2016–2026.

The earliest study in this review dates back to 2016, and publication activity has grown markedly since then. Although the search covered publications from 2015 onward, none of them met the inclusion criteria. Annual output remained low until 2020, after which it rose sharply (Figure 2a). More than three-quarters of the reviewed studies (82%) appeared since 2020, reflecting the recent acceleration of deep learning.

Corresponding author affiliations were identified for most of the studies (133 of 141), spanning 33 countries. Figure 2b presents this geographic distribution along with the trends in publication over time. China accounts for the largest share by a wide margin, followed by the United States, with several other countries contributing smaller numbers. The remaining studies are distributed across a long tail of additional countries, including Peru, Brazil, and Mexico from Latin America. This concentration broadly reflects the scale of postharvest engineering and precision agriculture research capacity in leading countries. The fact that these three Latin American countries each contribute only a single study points to a clear opportunity for the region to engage in deeper and more sustained research rather than one-off contributions.

### 3.2. Keyword Co-Occurrence Analysis

**Figure 3:**
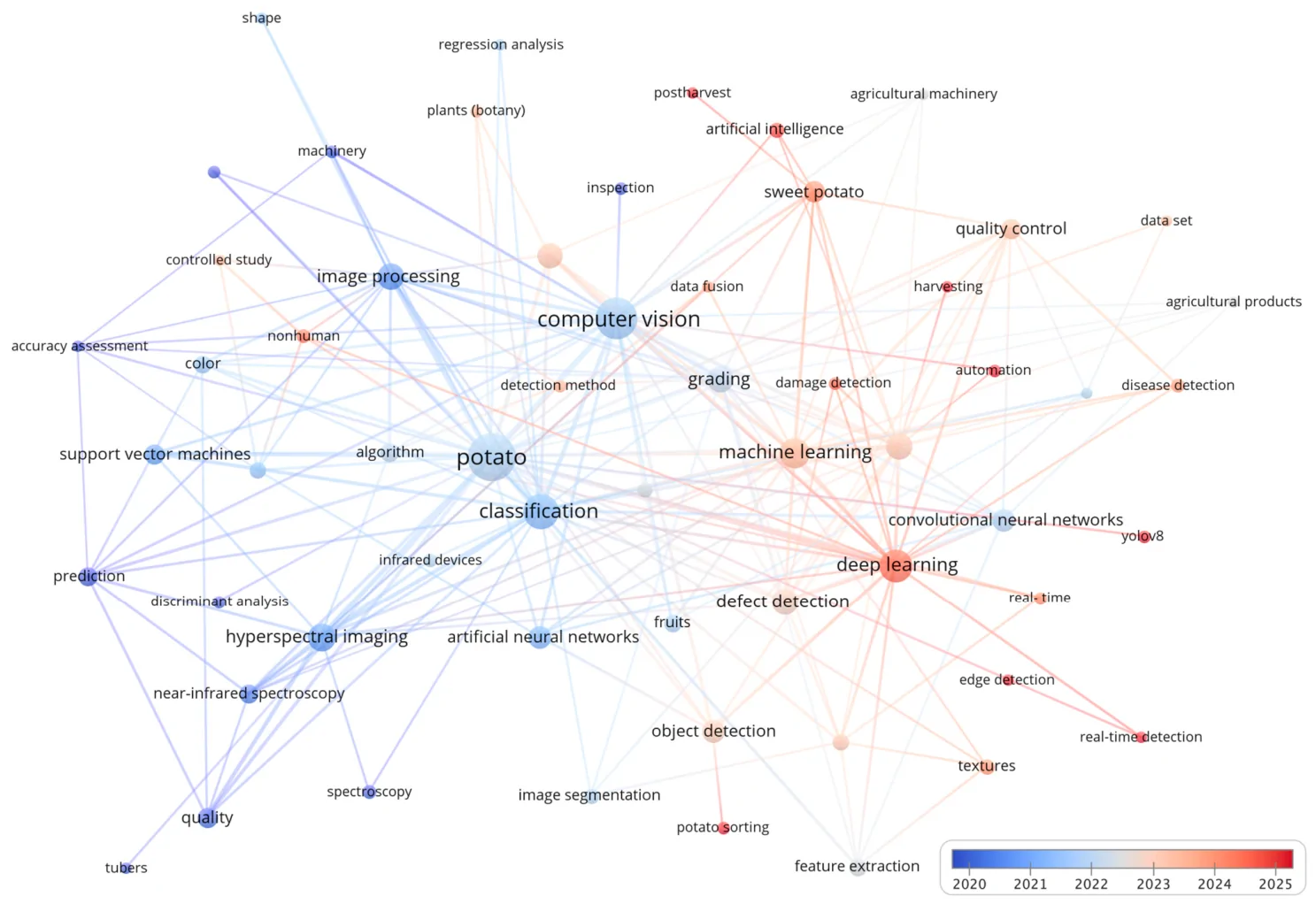
The VOSviewer keyword co-occurrence network of the 59 author keywords (occurring in ≥4 of the 141 included studies) retained after synonym normalization; node size denotes occurrence frequency, and node color denotes the average publication year of the studies using that term.

To show how research themes have changed over time, Figure 3 presents a co-occurrence network of 59 author keywords (see Section 2), each appearing in at least 4 studies. Node size reflects term frequency, while node color indicates the average publication year (2020–2025). The network reveals central themes, emerging topics in orange and red, and a strong integration between traditional sensing methods and modern computational technologies.

The network highlights a temporal shift in the field. Earlier studies represented in blue are associated mainly with conventional computer vision, image processing, hyperspectral and spectroscopic sensing, near-infrared spectroscopy, support vector machines, and quality prediction. More recent work, indicated by orange and red nodes, is increasingly centered on deep learning, CNNs, object and defect detection (including yolov8), real-time inspection, automation, and post-harvest applications. The network also suggests a gradual diversification from the central potato focus toward sweetpotato and other related agricultural variables.

### 3.3. Distribution by Crop Species

Table 2 shows how the included studies are distributed across crops, which frames the crop-by-crop analysis in Section 8. Potato (*Solanum tuberosum*) dominated the reviewed literature, accounting for 77.3% of the included studies. Sweetpotato (*Ipomoea batatas*) was a distant second with 17.0%, followed by cassava (*Manihot esculenta*, 2.1%) and carrot (*Daucus carota*, 1.4%). The remaining four species, namely oca (*Oxalis tuberosa*) [37], water chestnut (*Eleocharis dulcis*) [38], porang (*Amorphophallus muelleri*) [39] and sugar beet (*Beta vulgaris*) [40], are each represented by a single study (Table 2). This skew reflects the prominent global economic and research position of potato but also highlights that most minor crops, several of which are major regional staples, remain covered by only one proof-of-concept study each.

### 3.4. Distribution by Inspection Task and Technique Family

To allow a clearer synthesis, the reviewed studies were grouped into nine postharvest inspection tasks and three technique families. Image processing refers to classical, rule-based algorithms. Traditional machine learning refers to models trained on handcrafted features, such as support vector machines or random forests. Deep learning refers to neural networks, such as convolutional neural networks, that learn features directly from data. Table 3 cross-tabulates the reviewed studies by inspection task and technique family. Because many studies combine multiple technique families, the three method columns are not mutually exclusive.

As noted in Table 3, external defect and damage detection is the most frequent task, and deep learning has gained the strongest foothold, led chiefly by YOLO-family detectors [41,42]. This preference is driven by the need for real-time sorting and the difficulty in handcrafting features for surface blemishes, bruises, and cracks [43]. Disease and pest detection shows a similar preference for DL, although plain convolutional classifiers rather than detectors are preferred [44,45]. Foreign-object detection, that is, separating tubers from soil clods, stones, and plant debris, instead favors YOLO-family detectors for similar real-time reasons [46,47].

Sorting and grading stand out as the tasks where classical image processing still prevails, chiefly through thresholding and morphological operations [48,49]. This is largely because size, shape, and weight can be measured without training data, although deep learning is slowly entering this space for quality grading.

Field-level phenotyping, counting, and yield estimation are more balanced. Classical methods mainly rely on thresholding [50,51] and morphological operations [14,52], typically alongside photogrammetric reconstruction and depth measurements [53], while DL leans toward instance segmentation [54].

Non-destructive internal-quality estimation stands out as the task in which traditional ML methods hold the clearest lead. Studies in this category typically use spectral signatures from hyperspectral imaging, in some cases complemented with NIR spectroscopy, to predict reference chemical assays [12,55], an approach that has proven reliable without the need for large datasets.

The remaining tasks, including freshness and storage quality estimation, sprouting and greening monitoring, and variety identification, have fewer studies and no clear technical dominance.

Read across the rows of Table 3, the choice of technique family follows the nature of the task. Deep learning predominates where appearance is variable or objects must be located, as in external defect detection, disease and pest detection, and foreign-object detection. Image processing remains the most frequent family where size and shape can be measured directly, as in sorting and grading, and traditional machine learning leads where spectra are mapped to continuous chemical attributes, as in internal-quality estimation.

The shift toward deep learning is nevertheless marked. Its use has grown steadily across the review period, from a minority of studies in the earliest years to a clear majority in the most recent ones. This growth documents the adoption of these methods more clearly than it demonstrates a performance advantage, because the studies differ in crop, dataset, acquisition conditions, and evaluation protocol, and the few direct comparisons give mixed results. The field is therefore not moving through a simple replacement of classical methods by deep learning but toward approaches chosen for the task, the available data, and the deployment constraints.

## 4. Multi-Source Data Acquisition Systems

Any computer vision pipeline begins with sensing because the quality of the acquired signal determines what can be detected or measured. Table 4 summarizes the sensing modalities reported across the reviewed studies, along with their typical devices and representative studies.

### 4.1. RGB Imaging Systems

Conventional RGB imaging appears in most of the reviewed studies, either alone or alongside another technique (Table 4). These studies fall into three main deployment contexts. In industrial settings, cameras are mounted directly on packing or grading lines on conveyor belts [56]. In the laboratory, cameras are placed inside lightboxes or on fixed rigs with controlled illumination to standardize imaging conditions [58]. A smaller but growing number of studies have taken cameras into the field, mounting RGB sensors, sometimes with depth cameras, directly on harvesting machinery to capture tubers as they emerge from the soil [18]. Across these contexts, the hardware ranges from low-cost industrial sensors to consumer-grade DSLRs, and the operational throughput typically determines the resolution and frame rate rather than the specific inspection task.

### 4.2. Hyperspectral, Multispectral, and Near-Infrared Spectroscopic Systems

Spectral sensing is the second most common family of acquisition systems in the reviewed literature (Table 4). Hyperspectral systems are typically pushbroom line-scan cameras operating in the visible-to-NIR range. They are mounted above a motorized translation stage inside a light-sealed enclosure with halogen illumination. This configuration has remained essentially unchanged from the earliest study in this review, a dual VIS-NIR/SWIR camera system published in 2016 [59], to more recent portable units such as the SOC710-VP [60,78] and the Pika XC2 [13]. These systems are used almost exclusively for non-destructive internal-quality estimation, predicting attributes such as starch [12] and moisture [79] content from the spectral signature of the tuber surface rather than its visible appearance.

In five of the reviewed studies, point NIR spectrometers appear alongside camera-based systems, either RGB [20,21] or hyperspectral [22,63,65], as an interactance, reflectance, or transmittance measurement paired with a chemometric regression or classification model. The spectral measurement adds compositional information that the image alone does not capture, so in these pipelines NIR complements, or is compared with, the image-based acquisition rather than serving as the only modality.

In the reviewed studies, multispectral imaging, which trades fine spectral resolution for a small number of discrete bands, is comparatively rare in the reviewed studies. One example is the pairing of an interactance camera with a reflectance camera to detect nematode galls in harvested sweetpotato roots [70]. Another method uses a snapshot mosaic multispectral sensor to detect and classify multiple potato defect types [71].

### 4.3. Three-Dimensional, Depth and Other Modalities

Turning to the less common modalities, a three-dimensional or depth-sensing component was incorporated in 17 studies. These studies used a heterogeneous set of depth sensors and 3D acquisition configurations. The Intel RealSense L515 appears in four studies from what appears to be the same research group using slightly different acquisition protocols [9,10,66,80], most often to non-destructively estimate tuber volume or geometric dimensions. Instead, one study reconstructs three-dimensional tuber shape from multiple synchronized RGB cameras using structure-from-motion [14].

Thermal imaging appears in a handful of studies, typically to detect subsurface bruising or moisture-related temperature differences not visible in the RGB channel, as in an infrared thermographic camera used to assess postharvest deterioration [15].

Only one study ventures beyond conventional imaging for internal defects. It uses a computed tomography scanner alongside magnetic resonance imaging to visualize impact-induced internal bruising in potato tubers and reconstruct the damaged tissue in three dimensions, validating the result against physical cross-sections [17]. This study relies on a conventional RGB-based classifier for external defects, serving as a proof-of-concept comparison rather than an established acquisition pathway.

A small cluster of studies used laser backscattering imaging or biospeckle analysis. Rather than capturing a conventional intensity image, these techniques illuminate the tuber with coherent laser light and characterize either the intensity profile of the backscattered light or the speckle pattern activity it produces. One study used this approach to assess the storage quality of sweetpotato from the backscatter pattern alone [16]. Another study applied the same signal to discriminate potato tubers from clods and stones in a production environment [77]. The third method combines it with a classifier to grade the potato storage condition [69]. A fourth study compared it directly with a conventional visible-light camera for identifying skin-peeling lesions, finding that the more common modality is more effective for lesions stored for at least one day, whereas biospeckle performs better on fresher injuries [7]. Although still limited in number, these studies indicate that the modality landscape for tuber inspection remains open to innovation.

Taken together, RGB and spectral imaging account for most acquisition modalities used in the reviewed studies. The remaining modalities, including 3D and depth sensing, multispectral imaging, thermal imaging, X-ray and computed tomography (CT), and laser backscattering or biospeckle analysis, collectively cover a smaller but methodologically diverse minority of studies. Each is chosen to access an attribute, surface geometry, subsurface temperature, or internal structure that conventional RGB imaging cannot directly capture.

### 4.4. Acquisition Environment and Standardization

The acquisition environment varies widely across the reviewed studies and is at least as important as the sensor itself for model performance. Most studies (*n* = 94, 67%) captured images under controlled laboratory conditions with fixed lighting, a uniform background, and a static or slowly moving tuber. This simplifies segmentation but limits the reported performance’s direct transferability to packing lines or field conditions. Twenty-eight studies (20%) acquired images directly in the field or during active harvesting operations, where illumination, soil or foliage occlusion, and vibration are far less controllable, as in a harvester-mounted system whose real-time detection speed must satisfy fixed conveyor-belt timing constraints [81]. In addition, seven studies (5%) reported acquisition inside a commercial packing or processing facility, working with production-scale equipment and industrial partners [82]. The acquisition environment is not specified for the remaining 12 studies (9%). This imbalance toward controlled laboratory conditions is a recurring limitation, as models validated under such settings may not perform reliably under the more variable commercial operation conditions.

The acquisition environment followed the task and the sensor. Field acquisition concentrated on tasks carried out at harvest, such as detecting soil clods and stones on the harvester conveyor [46,83] and, to a lesser extent, field phenotyping, counting, and yield estimation [53,54], whereas sorting [49], grading [48], and internal-quality studies [12,55] were almost always laboratory-based. All field-based studies on foreign material used deep learning, so this setting was more common among deep learning studies than among the others, which partly reflects the task rather than the method. All but one of the hyperspectral studies were laboratory-based, with authors citing the need for controlled illumination and calibration, instrument cost, and processing time as limitations [12,22,84,85]. Field acquisition grew over time, while the laboratory share fell, although laboratory capture remained the most common setting in every period. These patterns are descriptive, and because the periods coincide with the spread of deep learning, they do not establish causes.

Surface coverage was also limited. Only 15 studies (11%) imaged tubers from more than one side, using rotating roller conveyors [5,9,10,80], 360° rotation [19], laser triangulation [51], fixed multi-view setups [14,49,86,87,88,89], or two-sided spectral scans [61,90,91]. These approaches allow different parts of the tuber surface to be captured. However, the number of views was not specified in the remaining studies, most of which appear to have used a single image or scan per tuber. This may leave defects on hidden surfaces undetected.

Image acquisition during movement was reported in 26% of the studies, using conveyors, rollers, or similar systems. Most involved laboratory prototypes designed to simulate production lines, operating at speeds of 0.15–0.5 m/s [92,93]. Only a few studies evaluated their systems on commercial lines [20,43,94], where speeds reached 1 m/s [94]. Other studies mounted cameras on harvesters [14,81,83], where dirt, vibration, and variable illumination complicate image acquisition. Overall, most evaluations under moving conditions were conducted in laboratory settings rather than commercial environments.

### 4.5. RGB vs. Spectral Imaging: A Summary of Trade-Offs

As described in the previous sections, RGB and spectral imaging together account for most of the studies in this review (Table 4). They are often presented as alternative choices, so clearly stating their respective trade-offs is useful.

RGB imaging offers practical advantages. Sensors are inexpensive, ranging from consumer smartphone cameras to industrial units, and their acquisition is fast enough to support real-time use on conveyor lines and harvesters. This combination of low cost and speed is reflected in the dataset sizes observed across studies, with one case exceeding 200,000 images after augmentation [95]. The main limitation is that RGB imaging mainly captures surface-visible information. An RGB image records only visible-light reflectance from the tuber surface; thus, it cannot assess attributes such as starch content, internal bruising, or blackheart, which require light to penetrate beneath the surface. Most studies on non-destructive internal-quality estimation in this review rely on a spectral or depth modality, and the RGB-based oca study [37] is an exception.

Hyperspectral imaging inverts this trade-off. By measuring reflectance across many narrow bands rather than three broad ones, it resolves the chemical and structural information that is not visible. This explains their dominance in internal-quality and physiological-disorder tasks. This informational advantage comes at a cost. Hyperspectral sensors and their scanning stages are more expensive and slower to operate, which is reflected in the smaller sample sizes of spectral studies, with as few as 96 physical scans in one case [12,96], and in their near-total absence from real-time, line-deployed contexts.

The evidence supports a task-dependent choice over a universally superior modality. When the target attribute is visible at the surface and throughput is important, RGB is preferred. When the target attribute is not visible at all, hyperspectral or NIR (as a complement of RGB images) is adopted, and its higher cost and lower throughput are accepted as the price of that access.

### 4.6. Most Commonly Recurring Specific Acquisition Devices

Several specific devices have been reported across multiple independent studies. The most common is a Canon-branded camera, which was used in ten studies (7%) across laboratory, harvester-mounted, and multi-camera setups [14,18,41,58,97,98,99,100,101,102]. The Specim ImSpector V10 or V10E spectrograph is used in nine studies (6%) as the core component of many custom-built hyperspectral systems [12,22,55,64,65,90,103,104,105]. Six studies (4%) used Basler industrial cameras across various models for conveyor-mounted defect detection [11,26,56,76,106,107]. The Intel RealSense L515 depth camera appears in four studies (3%) [9,10,66,80], and Hikvision industrial cameras appear in five studies (4%) [20,47,108,109,110]. No other specific device model recurs across different research groups in the remaining studies.

### 4.7. Processing Hardware and Deployment Architectures

Most studies reported the hardware used for model development, but fewer described the platform used for real-time inference. GPU-based processing was reported in nearly half of the studies, mainly using NVIDIA hardware. These resources were generally associated with model training or offline evaluation.

The distinction between training and inference hardware was explicitly described in fewer than ten studies. In these cases, training was performed on a workstation, server, or cloud platform, while inference was transferred to an industrial computer or an embedded device. This configuration was reported in studies using Jetson platforms and industrial computers for field or conveyor operation [5,46,81,111,112,113].

Edge and embedded hardware appeared in 18 studies (13%). Jetson boards were used mainly for local inference, while Raspberry Pi systems and microcontrollers also appeared in low-cost prototypes and control systems [45,83,88,114,115]. However, these studies did not always report latency, memory use, power consumption, or sustained operating performance.

PLC-based or equivalent control integration was identified in approximately ten studies. These systems connected the vision output with conveyors, pneumatic cylinders, motors, or rejection mechanisms [47,73,92,107,112]. This pattern was more common in sorting and separation applications than in studies focused only on image classification.

The association with the agricultural context was clearer than the association with crop species. Laboratory studies generally relied on workstation or GPU processing, whereas field and harvesting applications more often used local or embedded processing. PLCs and industrial control components were mainly found in systems where the model output triggered a physical action. Since potato represented most of the reviewed studies, the apparent concentration of specific hardware configurations in potato research should be interpreted cautiously. Table 5 summarizes the processing hardware and deployment patterns identified across the reviewed studies.

## 5. Classical Digital Image Processing Techniques

Table 6 summarizes the classical image processing techniques identified across the reviewed studies, together with their typical purpose and representative studies. As shown in the table, thresholding, morphological operations and grayscale conversion form the most common preprocessing backbone, whereas texture and shape descriptors appear more selectively.

### 5.1. Preprocessing

Most studies apply one or more basic preprocessing steps before any segmentation or feature extraction. These include cropping to a region of interest, resizing to a fixed input size, denoising, and placing the tuber against a uniform background. Background separation is made explicit in some studies, as shown in Table 6. In most cases, this is achieved physically by imaging the sample against a solid black or white surface rather than through algorithmic segmentation, as in a sweetpotato grading pipeline that cleans each sample, positions it on a solid black background, and resizes and normalizes the image before passing it to a classifier [3].

Conversion to grayscale is also common. This method discards color information that is not required for later thresholding or edge-detection steps. For example, before any segmentation is attempted, a carrot shape-grading pipeline first reduces an RGB image to a single channel using a linear combination based on the Ohta color space (a set of three orthogonal color features designed to approximate human perception) [121]. These preprocessing choices are not merely convenient. Even studies built around deep-learning classifiers or detectors almost always retain a classical preprocessing stage.

### 5.2. Color-Space Transformations and Color-Based Analysis

Although RGB is the default image capture representation, many studies have converted to a different color space before segmentation or feature extraction. HSV is the most common alternative, as shown in Table 6. Its advantage lies in separating color from brightness, making thresholding more robust to uneven illumination than working directly in RGB. A colorimetric analysis of oca tubers, for example, tracks browning and other surface changes during storage using HSV descriptors together with a fractal-dimension texture measure [37]. The perceptually uniform L*a*b* (CIELAB) space appears less frequently, typically used when subtle color differences need to be quantified rather than simply classified, as in detecting early discoloration associated with defects.

### 5.3. Segmentation: Thresholding and Morphology

Thresholding is the most widely used classical segmentation strategy, appearing in approximately one-third of studies (Table 6). Of these, a smaller subset applied Otsu’s method to automatically select the threshold rather than manually fixing it. A representative example estimates the length-to-width ratio of potato tubers by first isolating each tuber with Otsu-based binary thresholding and then refining the mask with morphological opening and erosion to remove small artifacts [117]. A similar combination of fixed-channel thresholding and morphological clean-up supports a real-time potato size-grading system built around a linear support vector machine [48].

Morphological operations (erosion, dilation, opening, closing) are common beyond thresholding-based pipelines, serving as general-purpose tools to fill small holes, separate touching tubers, or smooth segmentation boundaries from any upstream method, including deep learning. For example, repeated erosion and dilation fill small gaps in depth images before potato quality classification with a CNN [68], while opening and closing refine lesion masks from a deep-learning-based potato scab detector [108].

Watershed segmentation, by contrast, is rare among the studies analyzed, found in only one study, where it is combined with Otsu thresholding, Canny edge detection, morphological operations, and a U-Net model within a mobile-phone measurement pipeline [123].

### 5.4. Feature Extraction and Descriptors

Once a tuber has been segmented, classical pipelines typically summarize its appearance with a small set of hand-crafted descriptors rather than passing raw pixels to a classifier. As noted in Table 6, texture descriptors based on the gray-level co-occurrence matrix (GLCM) are the most common choice. For example, one study classified potato skin roughness into four severity levels using texture statistics alone, without any deep-learning component [99].

Shape-based descriptors support tasks in which the defect or grading criterion is essentially geometric. For instance, a carrot grading pipeline locates fibrous roots and cracks by detecting concave points along the root’s convex hull [121]. Fractal-dimension descriptors, which quantify the geometric irregularity of a surface, are markedly rare, found in only a handful of studies. One of them is the oca storage study that tracks browning and other surface changes during storage [37].

Across these techniques, thresholding, morphological clean-up, and grayscale conversion form a near-universal preprocessing backbone, touching roughly one-third to one-quarter of the reviewed studies. Color-space transformations and texture or shape descriptors are applied more selectively to tasks in which color or geometry is the discriminating attribute. Fractal-dimension and Fourier shape descriptors, by contrast, remain confined to isolated proof-of-concept studies rather than established practice. This pattern—a small set of general-purpose operations reused almost everywhere and a longer tail of specialized descriptors reused rarely—recurs in how deep learning architectures are adopted across the reviewed studies.

## 6. Machine Learning and Deep Learning

### 6.1. Traditional Machine Learning

A substantial subset of the studies did not use deep learning. Instead, classical or spectral features are paired with traditional statistical classifiers. Support vector machines (SVM) are the most common, used in 26 studies (18%). They range from a linear-kernel SVM that grades potato tubers by size on a conveyor line [48] to studies where SVM serves as a baseline against a purpose-built model.

Partial least squares (PLS) appears in 21 studies (15%). It is the default choice for relating a hyperspectral or NIR reflectance signature to a continuous quality attribute, such as starch or moisture content, as in the potato starch-prediction pipeline that combines PLS regression with a competitive variable-selection step [12].

Shallow neural networks or multilayer perceptrons (MLP) have been reported in 14 studies (10%), for example, when combined with linear and quadratic discriminant analysis to classify potato tubers based on their color and texture features [1].

Random forest (RF) has been used in eight studies (6%). k-nearest-neighbors (kNN) appears in seven studies (5%), illustrated by a cassava study that compares kNN against linear discriminant analysis, SVM, a decision tree, and an ensemble classifier on thermal-image features [4]. Extreme learning machines (ELMs) appear in two studies (1%).

These traditional approaches remain competitive because they require far fewer labeled examples than deep networks, an advantage given the small size of many datasets in the reviewed studies. An SVM maps features into a space and finds the hyperplane that separates classes with the widest margin. When classes are not linearly separable, a kernel function projects the data into a higher-dimensional space. The same logic applies to regression in the form of SVR. Because the number of parameters an SVM must learn scales with support vectors rather than image dimensionality, it performs well on the small feature-based datasets that dominate the reviewed studies [48,57,63,122].

### 6.2. Convolutional Neural Networks for Classification

For whole-image classification, CNNs dominate. The reviewed studies relied on standard backbones from general image recognition. ResNet appears in 13 studies (9%), Inception/GoogLeNet in 6 (4%), VGG in 6 (4%), MobileNet in 5 (4%), and EfficientNet in 5 (4%). Most are adapted via transfer learning rather than training from scratch.

A representative comparison evaluates six backbones (VGG16, VGG19, ResNet18, ResNet50, ResNet101, and a modified AlexNet) for classifying potato tubers by condition. Shallow ResNet18 has the best overall accuracy despite its small size [75]. The input is not RGB but an infrared thermogram remapped into three channels; thus, the comparison runs on thermal rather than RGB statistics [75].

A sweetpotato root-knot nematode gall detection study fine-tunes ResNet101, ResNet50, ResNet18, MobileNetV2, InceptionV3, and NASNetMobile on spectral imaging data [70]. A cross-domain potato defect classification study built around GoogLeNet adds an adversarial domain adaptation component, enabling the transfer from one setup to different lighting or camera conditions [129]. Most classification studies compare standard backbones rather than proposing new architectures. This contrasts with object-detection studies in which novel designs are more common.

### 6.3. Object Detection Architectures

**Figure 4:**
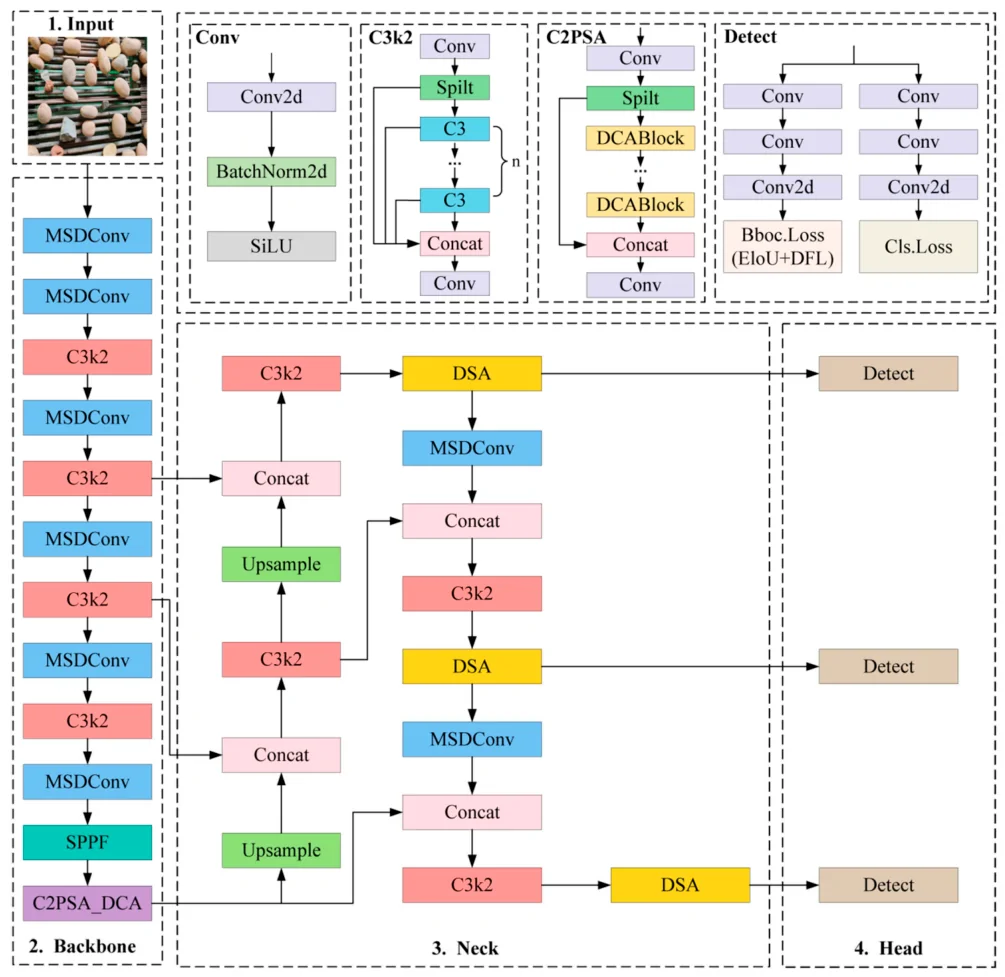
Architecture of a representative improved YOLOv11 object detection network comprising a backbone with a multiscale depthwise-separable convolution module (MSDConv), a feature-fusion neck, and a multiscale detection head. Colors indicate different layer types in the YOLO architecture, showing their use throughout the model. Reproduced from Ref. [46] under the Creative Commons Attribution (CC BY) 4.0 license.

Unlike region-proposal detectors, YOLO treats detection as a single regression problem. The input image is divided into a grid; one convolutional pass simultaneously predicts candidate bounding boxes, an objectness confidence score per box, and a class-probability distribution per grid cell [41]. Figure 4 shows a representative YOLO-based architecture, illustrating how the backbone, neck, and detection head are organized within this single-pass scheme.

A backbone extracts feature maps. A neck, typically a feature-pyramid or path-aggregation structure, merges features from multiple resolutions to detect both small and large objects in one pass. A detection head converts merged features into box coordinates, confidence scores, and class labels, filtered through non-maximum suppression [46].

This single-pass design gives YOLO its speed advantage over two-stage detectors, such as Faster R-CNN [41], explaining its presence in real-time, conveyor-line, and harvester-mounted contexts. The attention modules, multiscale fusion blocks, and pruning found across YOLO variants are modifications layered onto the same basic scheme, not fundamental changes. Figure 4 shows a representative example of a backbone with an added MSDConv, a feature-fusion neck, and a multiscale detection head. This illustrates how attention or fusion components are grafted onto the standard backbone-neck-head structure.

Object detection was the most frequent deep-learning task in the reviewed studies. Within it, YOLO dominates. Some versions of YOLO, from early releases through YOLOv11, YOLOv12, and YOLO26, appear in 31 studies (22%). Almost all are adapted with attention or multiscale-fusion modules rather than being applied off the shelf.

A YOLOv11-based detector with directional attention and multiscale convolutions sorts potato tubers on a conveyor into four categories with an mAP@0.5 of 97.7% [46]. After structured channel pruning, a pruned YOLOv8n variant with a lightweight HGNetv2 backbone and a re-parameterized detection head reaches a mean average precision (mAP) of 95.3% across seven external potato defect categories [41].

**Figure 5:**
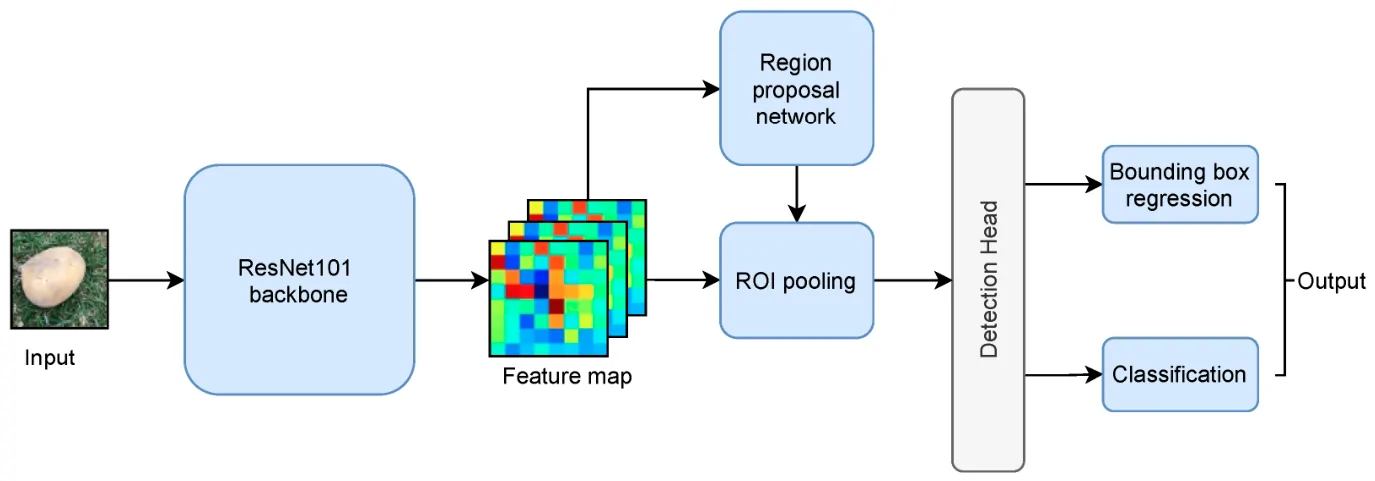
Faster R-CNN architecture with a ResNet101 backbone, one of three region-based detectors (alongside SSD Inception V2 and R-FCN ResNet101) benchmarked for potato surface-defect detection. Reproduced from Ref. [26] under the Creative Commons Attribution (CC BY) 4.0 license.

Region-based detectors, such as Faster R-CNN, are rare, appearing in only six studies (4%). One transfer-learning study fine-tuned and compared three deep-learning object detectors (Single Shot Detector (SSD) Inception V2, R-FCN (Region-based Fully Convolutional Network) ResNet101, and Faster R-CNN ResNet101) for potato surface defect detection on a processing line [26]. Figure 5 shows the best-performing architecture, Faster R-CNN with ResNet101, illustrating the two-stage region-proposal-then-classification design that distinguishes it from single-pass YOLO.

Transformer-based end-to-end detectors are emerging but still uncommon, found in three studies (2%). A wavelet-attention variant of the real-time detection transformer (RT-DETR) reaches an mAP@0.5 of 95.0% for classifying material on a potato harvesting line in the field [93]. A related RT-DETR model applied to water chestnut adds a multiscale dynamic-attention module and a morphology-aware loss function [38]. A third RT-DETR variant replaces the backbone with a lightweight ConvNeXtV2 network and adds a BiFormer attention module to classify potatoes, clods, and stones on a harvester conveyor, raising AP@0.5 from 93.3% to 96.3% while running on a GPU at 70 FPS [83].

One study departs from both detector families. Instead of proposing candidate boxes first, it predicts each object directly from its estimated center point, an anchor-free CenterNet-based design, and adds an ACmix attention module on top; it reports 97.5% accuracy when applied to separating potato tubers from soil and stone impurities [92].

### 6.4. Semantic and Instance Segmentation

Pixel- or instance-level segmentation is reported far less often than detection or whole-image classification. This reflects both the higher annotation cost and the fact that many grading and sorting tasks can be solved using a bounding box alone.

U-Net-based semantic segmentation appears in two studies (1%), including the mobile-phone tuber-measurement pipeline, where a ResNet34-encoder U-Net is combined with several classical processing steps [123]. DeepLabV3+ was used in a single study, where a ResNet50-backbone model performed three-class semantic segmentation of sweetpotato images and was paired with a separate Mask R-CNN instance-segmentation stage to isolate individual tubers, achieving a reported instance grading accuracy of 99.98% [82].

The Segment Anything Model (SAM) is used in a single study, not as the final classifier but as an intermediate segmentation aid. A potato sizing study segmented tubers using YOLOv8.1 and SAM before estimating the grade and mass from the resulting masks [88].

### 6.5. Attention Mechanisms, Transformers, and Lightweight/Edge-Optimized Models

Many studies do not use standard, off-the-shelf models. Instead, existing architectures are modified by adding attention mechanisms, transformer components, or compression techniques to meet practical deployment needs.

Attention modules appeared in 16 (11%). Examples include a convolutional block attention module (CBAM)-augmented YOLOv8s for potato surface defects [107], a multi-task YOLOv8 variant with multiscale attention and deformable convolution [42], and a dual-branch YOLOv8 detector that combines CBAM with a parallel-bottleneck module to grade potato scab severity [108].

Full transformer blocks are rare, found in four studies (3%). These include three RT-DETR-based detectors [38,83,93] and a Vision Transformer used alongside ResNet101 and EfficientNet-B3 for potato classification, with Gradient-weighted Class Activation Mapping (Grad-CAM) for interpretability [2].

Explicit model compression appears in only three studies (2%). These are the pruned HCRP-YOLO detector [41], a YOLO-based sweetpotato detector that combines Ghost convolutions with knowledge distillation [130], and a MobileNetV4-based classifier that applies structured channel pruning using the Automatic Training Once (ATO) method [3].

More broadly, 18 studies (13%) mention edge or embedded hardware, naming specific boards such as Raspberry Pi, Jetson, Arduino, or a Field-Programmable Gate Array (FPGA). However, most of these studies report only offline or bench-level validation on the target board, not sustained operation on a commercial line or harvester. Actual field or line deployment remains rare, indicating a small but consistent effort toward deployable systems rather than a mature practice.

### 6.6. Training Considerations: Augmentation, Transfer Learning, and Validation

Several training decisions recur across deep-learning studies beyond architecture choice. Data augmentation, including geometric transformations, such as rotation, flipping, and cropping, and in some cases synthetic samples from GANs, is explicitly reported in approximately 30% of the reviewed studies. The remaining studies either do not use it or do not report whether they do. This is important because many of the datasets in the reviewed studies are quite small. Synthetic data generation could help address this limitation but was rarely tested.

Transfer learning, where a backbone pretrained on a large general-purpose dataset is fine-tuned on tuber images, has been reported in at least 11 studies (8%). This includes the multi-backbone sweetpotato comparison [70] and the domain-adaptive potato classifier [129]. Transfer learning makes the standard-backbone classification strategy practical on datasets of only a few hundred to a few thousand images.

A fixed train/validation/test split is the more common practice for evaluation, used in 82 studies (58%). Cross-validation or leave-one-out validation is reported in 40 studies (28%). The remaining studies either validate against an independent reference measurement instead of a held-out split or do not clearly specify their protocol.

This distinction is important when comparing the performance across studies. A model evaluated with k-fold cross-validation and one evaluated on a single held-out split are not scored under strictly comparable conditions, a caveat to consider when synthesizing reported metrics. Table 7 lists a representative set of the deep-learning architectures discussed in this section by crop, inspection task, and best reported performance.

## 7. Applications by Inspection Task

This section organizes the reviewed studies according to the objective of the inspection. The studies fall into nine tasks, each with its characteristic crops, techniques, and metrics. Table 8 summarizes these tasks using representative examples. Each task is discussed in turn, and the closing paragraph draws out the recurring patterns.

### 7.1. Sorting and Grading by Size, Shape, and Weight

**Figure 6:**
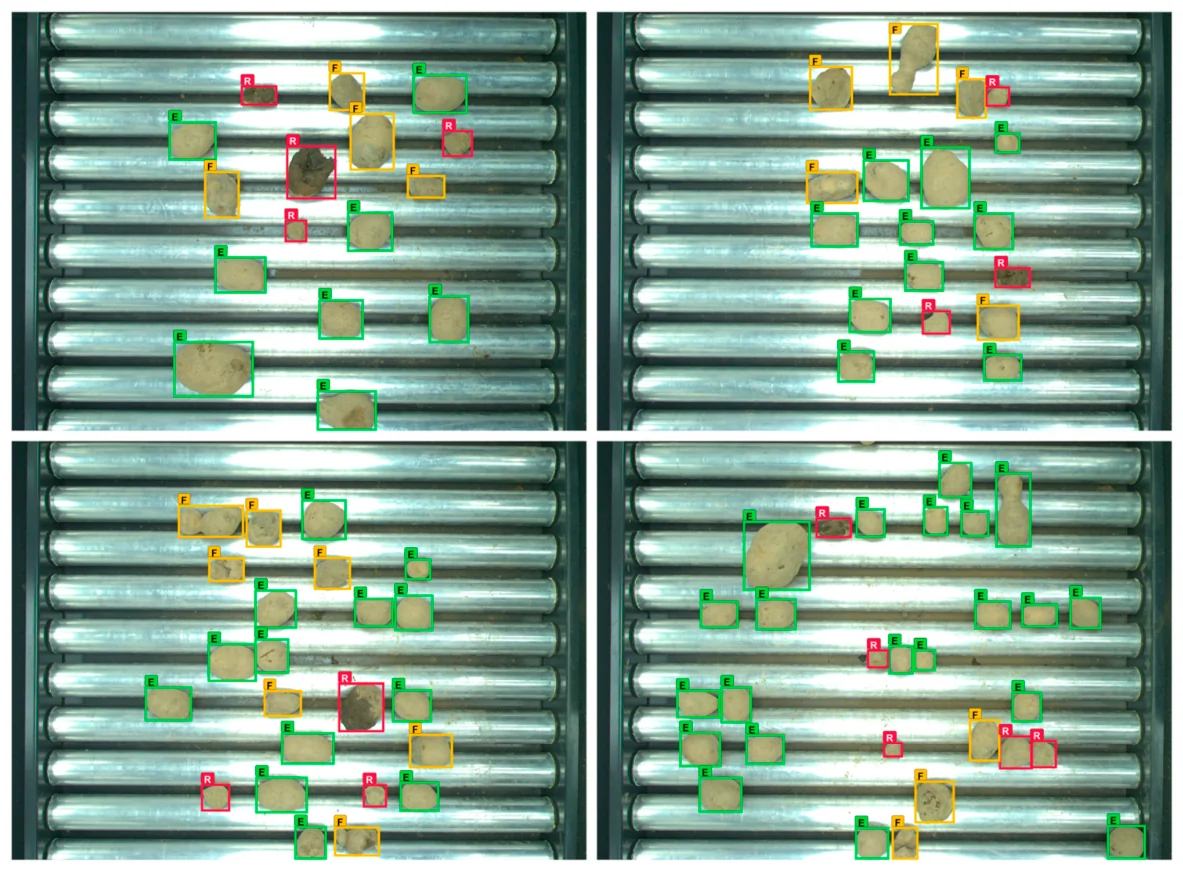
Representative output of a YOLO-based potato detector on a roller conveyor, classifying tubers as edible (green), feed-grade (yellow), or rotten (red) under the same-site test conditions. Reproduced from Ref. [11] under the Creative Commons Attribution (CC BY) 4.0 license.

Sorting and grading tubers according to physical dimensions is the most direct machine-vision task in the literature reviewed. A typical approach converts a segmented tuber silhouette into length, width, and estimated mass, and then assigns the tuber to a commercial size class. A linear SVM applied to a thresholded and morphologically cleaned potato image reaches 98.1% accuracy when separating tubers into size classes on a moving line [48]. A combined YOLOv8.1 and SAM segmentation pipeline estimated potato grade from tuber width with 100% classification accuracy and predicted mass from the segmented area with an R^2^ of 0.99 [88]. A third study grades tubers by market quality (edible, feed-grade, or rotten) with a YOLO-based object detector deployed directly on a roller conveyor, illustrating how this kind of real-time detection output looks in practice, as shown in Figure 6 [11].

Depth or 3D imaging supports the same task with direct geometric measurement rather than a 2D silhouette. A multi-view RGB-D system mounted over a roller conveyor estimates carrot width, length, volume, and mass with a mean absolute percentage error of 2% by aggregating several viewpoints of the same root [5]. Because the target variable in these studies is usually a continuous physical measurement rather than a qualitative judgment, sorting and grading are the tasks where classical geometric image processing is most directly competitive with deep-learning detectors.

### 7.2. External Defect, Damage, and Skin Disorder Detection

External defect detection covers a heterogeneous set of surface conditions, including cracks, cuts, skinning, bruising, and rough or scabbed skin. Detection-based approaches dominate when the defect must be both located and classified. A YOLO-based pipeline detects and classifies potato surface defects with an mAP@0.5 of 94.3% [111], whereas a related multiclass detector reaches 96.7% mAP@0.5 and maintains 94.0% accuracy when tested on a packing line [112].

Where the defect is diffuse rather than localized, whole-image or per-region classification is preferred. A texture-based classifier grades potato skin roughness into four severity levels from GLCM descriptors alone, reaching 87.5% accuracy without any deep-learning component [99]. A feature-based neural network classifier grades skin color and overall raw-material quality with 95.56% accuracy [136].

Several pruned or attention-augmented detectors, including HCRP-YOLO [41], have been developed specifically for this task. This shows that external defect detection is where much of the architectural innovation in object detection is concentrated.

A further group, using lightweight detectors tuned for onboard operation, targets this same task directly on the harvester rather than on a packing line. Examples include an early MobileNetV3-YOLOv4 detector separating damaged from intact tubers during harvest [18], a lighter YOLOv8 variant for real-time damage detection [113], a YOLOv4-tiny detector for sugar-beet mechanical-damage typing during harvest [40], and a non-neural alternative that flags damaged potatoes by their surface-temperature signature using active infrared thermal imaging with Otsu-style thresholding and Synthetic Minority Oversampling Technique (SMOTE)-balanced classification [73].

### 7.3. Disease and Pest Detection

Disease and pest detection differs from generic external defect detection in that the visual symptom must be attributed to a specific pathogen or pest rather than treated as a general quality defect. This typically requires a finer-grained, multi-class classification. Using ResNet101, a CNN-based classifier separates common scab-infected potato tubers from healthy ones with 99.5% accuracy [44]. A detection and severity pipeline built around the CBAM-augmented dual-branch YOLOv8 model introduced earlier localizes scab lesions with an mAP@0.5 of 81.9% and grades the severity into four levels [108].

Full disease classification pipelines directly applied to potato tubers report even higher accuracy. A single CNN-based classifier achieves 98.97% test accuracy across several disease categories [27]. Another study reached 100% accuracy on individual disease classes with an overall accuracy of 98.65% [140].

Hyperspectral imaging extends the same task to pathogens with no obvious external symptoms. A study targeting zebra chip disease in potato tubers reached 92% accuracy, sensitivity, and specificity from full-spectrum reflectance data using a relatively simple preprocessing and classification pipeline [85]. This illustrates that spectral information, not only spatial information, can support disease detection ahead of visible symptoms.

### 7.4. Sprouting, Greening and Physiological Disorder Monitoring

Sprouting and greening are physiological rather than pathological changes. Several of the reviewed studies specifically aimed to detect them before they became visible to the naked eye. A chlorophyll fluorescence imaging study detects the onset of greening in stored potato tubers under white light 1–2 days before a visible color change under white light. It uses a color-difference threshold of ΔE below 3.5 and achieves millimeter-scale spatial resolution [114].

Sprouting is addressed as a binary classification problem in a study that compares an ensemble-based classifier with and without a multi-channel CNN feature extractor. The CNN-based variant improves the F-measure from 0.905 to 0.951 [142]. A related study directly targeted the potato eye and classified each eye as pre-sprouting or already sprouting with 97.6% accuracy [103].

Chlorophyll and total glycoalkaloid concentrations, both physiological indicators linked to greening, are predicted as continuous variables from hyperspectral reflectance with a coefficient of determination of 0.971 [90]. This shows that the task can be framed as regression as readily as classification.

### 7.5. Non-Destructive Internal-Quality Assessment

Internal quality attributes, such as starch, sugar, and moisture content, and internal defects, such as blackheart, cannot be assessed from conventional RGB images. Instead, they rely on hyperspectral or multispectral imaging, or in some cases RGB imaging complemented with NIR spectroscopy. In the reviewed literature, they are evaluated strictly through spectrum-based prediction rather than any method that requires cutting the tuber. The starch content is the most frequently predicted internal attribute. The CARS-SVR pipeline predicts potato starch content with a prediction-set correlation of 0.93 [12].

Internal defects invisible from the surface are addressed directly in a study that combines external defect detection with a binary internal blackheart classifier. It reaches 98.2% accuracy on the internal-defect task while also detecting external defects with an mAP@0.5 of 89.2% [20].

The moisture of sweetpotato is predicted with an R^2^ of 0.972, and the texture-related consistency with an R^2^ near 0.85, using the spectral pipeline already discussed for its acquisition modality in Section 4.2 [13].

Because none of these attributes can be verified from the image itself, every study in this category depends on a destructive reference measurement to generate ground truth, even though the resulting model is applied non-destructively at inference time. In addition, the sensors used for these predictions were almost always validated in the laboratory, so internal quality is one of the tasks least documented outside controlled conditions.

### 7.6. Identification and Authentication of Variety/Cultivars

Distinguishing between cultivars of the same species is a narrow but recurring task, which is generally framed as closed-set image classification with a set of known variety labels. A multilayer-perceptron pipeline jointly classifies potato variety and turgor status, achieving a validation quality score above 0.97 [24]. A SqueezeNet-based classifier distinguishes potato varieties and further separates regular from mini-tubers, reaching 98% accuracy for its best-performing variety [100]. A third study reports perfect classification accuracy across the potato varieties in its dataset [128].

Because the number of varieties considered is usually small and the visual differences between them subtle, these studies tend to rely on lightweight, off-the-shelf CNN backbones rather than purpose-built detectors and attention modules developed for defect and disease detection. This suggests that variety identification is a comparatively easier, more standardized classification problem in the reviewed literature.

### 7.7. Freshness, Ripeness, and Postharvest Storage Quality Estimation

Changes in freshness and storage-related quality changes are assessed either as a categorical judgment, such as fresh versus aged or healthy versus deteriorated, or as a continuous estimate of storage time or age. A classifier that jointly identifies commodity type and freshness level achieves 92.3% accuracy with an Area Under the Receiver Operating Characteristic Curve (AUC) of 0.942 [143]. A separate study frames the same problem as both a binary freshness classification and a continuous age-regression task on Yukon gold potatoes, reporting 98.0% accuracy on the classification side [144]. A three-class condition classifier that distinguishes healthy, germinated, and rotten potato tubers reaches 88.1% accuracy [6]. The cassava deterioration-level classifier introduced earlier for its use of k-nearest-neighbors reaches 86.7% accuracy on thermal-image features [4].

Compared with sprouting and greening studies, which target a specific physiological cause, freshness and storage studies tend to treat quality decline as a single ordinal or binary outcome without necessarily attributing it to a specific underlying mechanism.

### 7.8. In-Field Phenotyping, Counting, and Yield Estimation

Studies in this category move the imaging system out of the laboratory or packing line and into the field or onto a harvester. They aim to count, size, or phenotype tubers under working conditions rather than under controlled illumination, consistent with the field-based subset of acquisition environments described earlier.

A harvester-mounted camera system directly detects and classifies potato tubers into three material categories on the moving equipment at real-time speeds of approximately 45–60 frames per second (FPS), supporting a pneumatic ejection decision [81]. A combined RGB-D and stereo-photometry rig measures potato width and height by discrete counting bands, keeping error within 10% for most measurements while also imaging above-ground foliage [53].

Regression-based phenotyping is also used. A hyperspectral system predicts the number of leaf primordia, an internal developmental trait, from potato foliage with a correlation of up to 0.96 [105]. This illustrates that in-field phenotyping in the reviewed studies extends beyond the tuber itself to canopy-level traits measured non-destructively.

### 7.9. Real-Time and Robotic/Industrial Sorting-Line Integration

A recurring deployment-oriented task among the analyzed studies is separating harvested tubers from foreign material, such as soil clods, stones, or plant debris. This must be done quickly enough to support a real-time sorting or cleaning line.

A color- and shape-based classification pipeline separates potato tubers from clods and stones with an accuracy of >98% [146]. A related image-based study using color and texture features reports 100% accuracy for an artificial-neural-network classifier under two- and three-way classification schemes [1].

A YOLOv5-based system on a potato-harvesting line separates tubers from foreign material with a detection rate of 95.2% while estimating each tuber’s mass and volume from its image-derived dimensions [95]. An RT-DETR-based detector applied to water chestnut achieves an mAP@0.5 of 94.9% for distinguishing chestnuts from surrounding clods and background [38]. On a harvester conveyor, a lightweight RT-DETR variant with a ConvNeXtV2 backbone and a BiFormer attention module distinguishes potatoes from clods and stones, raising AP@0.5 from 93.3% to 96.3% while running at 70 FPS on a GPU [83].

Industrial-camera detectors, such as the YOLOv12-based potato detector, which reaches an F1 score of 0.97, complete this picture [56]. In this deployment context, inference speed and debris robustness are as important as raw classification accuracy. The same real-time logic extends upstream to the harvester-mounted system, which classifies tubers on the moving equipment at 45–60 FPS to drive a pneumatic ejection actuator [81]. This shows that the deployment constraints for stationary sorting lines apply equally to in-field inspections mounted on harvesters.

## 8. Applications by Crop

### 8.1. Potato (Solanum tuberosum)

Potato is, by a wide margin, the crop for which the reviewed studies offer the most complete picture. It is the only species represented in every one of the nine inspection tasks characterized in Section 7 and across every technique family discussed in Section 5 and Section 6, from classical Otsu and morphology grading pipelines to attention-augmented and pruned YOLO detectors.

This breadth spans sorting and grading by size [48], external defect and skin-disorder detection with a dedicated architecture such as HCRP-YOLO [41], disease detection for common scab [44], physiological monitoring for greening and sprouting [114,142], non-destructive prediction of starch content [12], variety identification [100,128], freshness classification [143], real-time breakage and impurity monitoring during harvest with MCDE-YOLOv11 [46], foreign-object classification of potatoes versus clods and stones via a lightweight RT-DETR variant [83], and harvester-mounted real-time deployment [81].

This concentration of effort plausibly reflects potato’s position as the world’s fourth most important food crop [1] and the correspondingly mature packing-line and precision-agriculture infrastructure already built around it. This provides researchers with access to industrial imaging setups, large sample sizes, and a well-established classification taxonomy of commercially relevant defects. Whether the methods validated on potato transfer to the far less-studied crops remains an open question.

### 8.2. Sweetpotato (Ipomoea batatas)

Sweetpotato is the second most represented crop in the reviewed literature, and despite its far smaller sample size than potato, it shows a comparably wide methodological range. At the classical-to-deep-learning boundary, a DeepLabV3+ and Mask R-CNN pipeline segments individual tubers and quantifies skinning damage with 99.98% instance grading accuracy [82]. A pruned, attention-augmented MobileNetV4 classifies external quality with channel pruning aimed at edge deployment [3]. Two complementary lightweight models, Ghost-YOLOv5 for field detection and a knowledge-distilled YOLOv5n-m for grading, reach 92.6% mAP for detection and 95.0% mAP@0.5 for grading [130].

A separate hyperspectral pipeline combining LARS-LASSO variable selection with SHAP-based interpretability predicts both vitamin C and reducing-sugar content with correlations above 0.96 [55]. A study applies HSV thresholding and point-cloud-based 3D reconstruction to estimate sweetpotato root volume directly, reaching an R^2^ of 0.958 [66], using the same RealSense-based acquisition setup.

This diversity, which is concentrated in a comparatively small set of studies, suggests that sweetpotato benefits from a handful of active, methodologically sophisticated research groups rather than the broad, industry-wide adoption seen in potato.

### 8.3. Cassava (Manihot esculenta)

The three cassava studies in the reviewed literature are notably heterogeneous in purpose. They span postharvest quality assessment, disease detection, and plant breeding support, rather than converging on a single dominant application, as potato and sweetpotato do. On the postharvest side, a thermal-imaging pipeline classifies root deterioration level from intensity and temperature features using several classical and shallow-learning classifiers, reaching 86.7% accuracy with its best model [4]. On the disease side, a transfer-learning EfficientNetB0 model classifies disease symptoms separately for cassava leaves, stems, and tubers, reaching 90–94% accuracy depending on the plant organ [45]. The third study departs from quality inspection altogether. It relies purely on classical thresholding in the hue, saturation, and brightness (HSB) color space to extract morphometric traits, such as root aspect ratio, to support genomic prediction for a plant breeding program [50]. This shows that not every computer vision application to these crops is aimed at grading or sorting.

Given the importance of cassava as a staple food providing the main calorie source for more than 800 million people worldwide [4], this comparatively thin and scattered evidence base stands out as one of the clearer gaps identified in the reviewed literature.

### 8.4. Carrot (Daucus carota)

The two carrot-only studies in the reviewed literature each rely on a different family of techniques. A multi-task YOLOv8 model performs object detection, instance segmentation, and geometric regression on a conveyor-mounted depth camera, estimating width, length, volume, and mass with a mean absolute percentage error below 2% through multi-view aggregation [5]. A fully classical pipeline grades carrots into three length categories plus a reject class using a convex-polygon and concave-point method, a Hough transform for shape analysis, and a Bayes classifier. It achieves 98.0% accuracy with only a 160-sample calibration set and no learned component [121].

### 8.5. Underexplored Species

Four species among the analyzed studies (oca, water chestnut, porang, and sugar beet) are represented by one study each. Together, they illustrate both the methodological range applied to root and tuber crops in general and how little of it has been repeated or extended for any single minor species.

A Peruvian study on oca tracks fractal dimension and HSV color changes during storage, finding a strong correlation between °Brix sugar content and a surface fractal dimension for one of the studied cultivars [37]. This is one of three Latin American contributions to the reviewed literature (alongside a Brazilian sweetpotato phenotyping study [101] and a Mexican potato quality study [76]), despite the region’s importance as a center of tuber-crop diversity.

In the reviewed literature, water chestnut is addressed with one of the most architecturally sophisticated detectors, a transformer-based RT-DETR variant with wavelet convolutions and a morphology-aware loss function, achieving an mAP@0.5 of 94.9% in separating chestnuts from surrounding clods [38].

Porang bulbils are classified as infected or healthy using Fisher’s discriminant analysis over color, GLCM texture, and geometric descriptors at 97.0% accuracy [39]. The mechanical damage classification of sugar beet combines an improved YOLOv4-tiny detector with a modified VGG-16 classifier, reaching 99% accuracy under normal illumination [40].

Taken together, these four studies demonstrate the feasibility of applying advanced detection, segmentation, and spectral regression methods to root and tuber crops beyond potato. However, each species is represented by only one study, highlighting the need for further research to validate and extend these findings. Future studies could also examine the transferability of these methods to morphologically distinct crops, such as cassava and carrot, and understudied Andean tubers, including ulluco, mashua, and oca.

## 9. Datasets, Benchmarks and Evaluation Metrics

### 9.1. Public vs. Proprietary Datasets and Data Availability

Data availability is one of the clearest and most consistent gaps identified in the reviewed literature. Only 5 of the 141 included studies (4%) report using an explicitly public or repository-hosted dataset, either in whole or in part, while the remaining 136 (96%) rely on images captured specifically for the study, described variously as self-collected, proprietary, or captured by the authors, with no indication that the resulting dataset was subsequently released. Even among the 5 exceptions, none constitute a dedicated, root- or tuber-specific benchmark of the kind common in other computer vision domains. One study draws on three named potato-disease datasets assembled from web and laboratory sources [148], one combines a filtered subset of the general-purpose Fruits360 dataset with additional web-crawled images to build a 2316-image potato dataset [6], one aggregates several multi-crop repositories from Kaggle and other sources to reach 25,988 sweetpotato-related images [25], one combines a public Kaggle cassava dataset with the authors’ own field images for external validation [45], and one benchmarks an ensemble of classical classifiers against CNN and Long Short-Term Memory (LSTM) models for vegetable disease detection using two public image datasets [149]. This means that besides these five exceptions, every quantitative comparison in the reviewed literature rests on a different, non-overlapping, and non-public set of images. Table 9 lists the five datasets, their crop, modality, reported size, and the studies that used them.

### 9.2. Annotation Practices and Dataset Size

The reported dataset sizes vary by more than four orders of magnitude across the reviewed studies. Where a usable image count could be extracted from the reported description, the median is approximately 1000 images, with a middle 50% of studies falling between roughly 280 and 3900. However, several spectral-regression studies are built on far fewer physical samples, such as a starch-prediction pipeline based on only 96 hyperspectral tuber scans [12,96], reflecting the higher per-sample acquisition cost of hyperspectral imaging. At the other extreme, an object-detection study expands 9906 originally captured images to 227,838 through data augmentation [95], and an industrial semantic-segmentation system processes on the order of 18,000 images per day of continuous commercial operation, analyzing more than 2.4 million individual tuber instances in total across the six production days monitored in the study [82], a scale unmatched by any other study in the reviewed literature. Manual annotation, which is most often performed with general-purpose tools such as LabelImg or LabelMe for bounding boxes and polygon masks, is mentioned only in passing in most studies. Details that would support reproducibility or an assessment of label quality, such as the number of annotators, any inter-annotator agreement check, or the annotation guidelines used, are rarely reported.

### 9.3. Evaluation Metrics Used Across Studies

Consistent with the classification- and detection-heavy technique mix, accuracy is the most frequently reported metric, appearing in 82 of the 141 studies (58%), followed by recall or sensitivity in 66 (47%) and F1 score in 38 (27%). Detection-specific metrics are less common overall but concentrated in the object-detection literature, with mAP reported in 22 studies (16%) and Intersection over Union (IoU) in 10 (7%).

The regression-oriented tasks rely on a largely disjoint set of metrics. R^2^ appears in 33 studies (23%) and Root Mean Square Error (RMSE) in 37 (26%). The ratio of performance to deviation (RPD), a chemometrics-specific indicator of whether a spectral calibration model is sufficiently accurate for practical use, is reported in 11 studies (8%). For example, an RPD of 2.69 for the CARS-SVR starch model [12] exceeds the commonly used RPD rule of thumb of 2 or higher for a usable predictive model, although the appropriate threshold is context-dependent.

Metrics that explicitly account for class imbalance, such as AUC, are rare, appearing in only three studies (2%). This is notable because several grading and defect detection tasks involve highly unbalanced class distributions between a dominant healthy or marketable category and much rarer defect classes.

### 9.4. Challenges in Comparability and Benchmarking

Taken together, the findings of this section make it difficult to justify a direct, quantitative comparison across studies. Each study builds on its own proprietary images, collected with its own acquisition device, lighting setup, and cultivar selection. Studies addressing the same nominal task report different results with only partially overlapping metrics. Evaluation protocols differ, with roughly half of the reviewed studies relying on a single fixed train/test split and approximately a quarter using cross-validation.

Therefore, a reported accuracy of 98% cannot be read as directly comparable across two studies unless their datasets, class definitions, and validation protocols are also comparable, which is rarely the case among the analyzed studies. This is the central methodological reason for adopting a narrative, qualitative synthesis rather than a quantitative meta-analysis. With no shared benchmark and only 4% of studies drawing on any public dataset (Table 9), pooling reported performance figures into a single summary statistic would overstate the comparability of the underlying evidence.

## 10. Trends, Challenges and Limitations Analysis

### 10.1. Data-Related Limitations

The limitations most frequently self-reported by the authors of the included studies concentrate on the data underlying their models, corroborating the near-total reliance on proprietary, self-collected imagery. Restriction to a single cultivar, region, or growing season is explicitly flagged as a limitation in some studies, including the directional-attention YOLOv11 detector, whose authors note that their data come from a single region and a single potato variety [46], and the RT-DETR-based harvester detector, similarly limited to one region and a narrow range of field conditions [93]. The RealSense-based sweetpotato volume-estimation study reported a reduced sample size, a single cultivar, and indoor-only image capture as limitations [66], a reminder that a single dataset’s constraints directly propagate into its reported results. More broadly, several studies explicitly identified the limited scale or diversity of their dataset as a constraint on generalization, as in a hyperspectral potato-seed external-defect detection study that combined 1D/2D CNNs with classical classifiers and described its own dataset as modest and called for validation under more varied environmental conditions [84].

### 10.2. Generalization and Robustness Under Real Operating Conditions

Beyond dataset composition, the authors frequently flag the gap between the controlled conditions under which most systems are developed and the variable conditions of real operation. Some form of lighting-related limitation has been reported in several studies, including an explicit lack of evaluation outside laboratory or otherwise controlled lighting for the carrot 3D-measurement system [5] and for a sweetpotato grading pipeline not yet tested with freshly harvested roots [9]. Occlusion and background complexity are also reported, including the mobile-phone tuber-measurement pipeline, whose authors note that complex backgrounds reduce accuracy and that their model may be biased toward simpler, less-cluttered backgrounds [123].

A broader gap between laboratory development and industrial deployment was also reported. For example, the pruned MobileNetV4 sweetpotato classifier was developed and evaluated entirely under controlled laboratory conditions without in-line industrial validation [3]. A lightweight RT-DETR-based potato, clod, and stone classifier reaches 70 FPS on a GPU but only 1.2 FPS when deployed on a Raspberry Pi 4B, an edge-device gap its own authors flag as limiting real-world use [83].

One study addresses generalization more directly than most. It deliberately splits its potato quality-grading dataset by geographic location, training on one site and testing on a different, previously unseen one, while benchmarking five YOLO versions (v8 through v11, plus the newly released YOLO26) and a Faster R-CNN baseline against each other. Even for its best-generalizing architecture, it reports a measurable drop from internal to cross-location performance (F1 of 0.9464 internally versus 0.9176 on the held-out location) [11]. This explicit cross-site evaluation protocol is rare in the reviewed literature, so its result is worth flagging as a lower bound on the generalization gap that other single-site studies do not directly measure. As mentioned earlier, field-acquired studies have become more frequent over time, which may indicate a gradual shift toward more realistic validation. However, this trend does not by itself demonstrate improved generalization, since cross-site and cross-condition testing remains rare. Field validation also remains concentrated in harvest-line tasks such as clod and stone detection, while spectral approaches are still validated almost exclusively in the laboratory. A recent review of sweetpotato quality also reports uneven sample coverage and limited testing across seasons, sites, cultivars, and instruments [36], which is consistent with the pattern found here across crops.

### 10.3. Computational Cost and Real-Time and Edge-Deployment Constraints

Few of the reviewed studies directly compared accuracy with computational cost. The available evidence suggests that good laboratory performance does not always translate to good field performance. A pruning study reported a substantial speed gain alongside only a minimal accuracy loss after compression [41]. This type of explicit trade-off analysis remains uncommon in the reviewed literature.

A more striking example is a size-grading system that achieves 98.1% accuracy in the laboratory but only 61–86% efficiency when deployed on a real line. The drop appears to be caused by capture delays, conveyor speed, irregular tuber movement, and mechanical issues with the ejector arms themselves [48]. This suggests that the classification metrics discussed in Section 9.3 only measure the model and not the full sorting system. A potato-defect detector similarly reports that accuracy falls at higher conveyor speeds, although it still runs sufficiently fast for real-time use [111]. Beyond these examples, studies that target lightweight or edge deployment rarely test their systems in actual field conditions. A review of hyperspectral imaging in potato reaches a similar conclusion for spectral systems, considering real-time modeling with full spectra currently unsuitable because of data volume and expecting practical applications to rely on multispectral systems [33].

### 10.4. Gaps in Reproducibility and Explainability

Reproducibility is limited by several partial gaps. Most studies use proprietary datasets that are not publicly available. Annotation procedures are rarely described in sufficient detail to be repeated. Evaluation protocols and metrics vary widely, making direct comparison difficult. Some studies report no limitations at all, which removes information that readers might otherwise use to judge how far a given result can be trusted or generalized. For the per-study list of self-reported limitations, see Supplementary Table S5.

A limited number of studies have used interpretability or feature-importance analyses. Some applied dedicated post hoc explanation tools such as SHAP or Grad-CAM, while others used variable-importance rankings for feature selection rather than to explain final model decisions. See Supplementary Table S5 for the full per-study breakdown. A hyperspectral sweetpotato study uses SHAP values to identify which spectral regions drive vitamin C and sugar predictions [55]. A vision transformer-based potato classifier uses Grad-CAM to visualize the image regions on which the model relies [2].

The scarcity of such interpretability steps suggests that, for most of the reviewed studies, a reported performance figure offers limited insight into why the model makes its decision. This may limit how confidently practitioners can apply the results to new cultivars or operating conditions.

Hardware reporting was also inconsistent. CPU, memory, inference platforms, and control interfaces were often omitted or described only briefly. This limits the reproduction of processing speed and makes it difficult to assess whether a model can be transferred from a laboratory computer to a field or industrial system.

### 10.5. Trade-Offs of Classical vs. Learning-Based Methods

Despite the deep-learning-dominated technique mix, direct within-study comparisons against classical or simpler traditional baselines are uncommon. A relatively small number of studies explicitly benchmark their proposed model against a classical image-processing pipeline or a traditional ML classifier trained on hand-crafted features. Where this comparison is made, the results tend to be mixed rather than uniformly favoring the more complex model. A sugar-beet damage classifier clearly outperforms a classical bag-of-visual-words pipeline built on histogram of oriented gradients (HOG) and Scale-Invariant Feature Transform (SIFT) descriptors [40].

However, a potato-sizing study comparing standard image processing, a machine-learning pipeline, and manual caliper measurement found that the accuracy of the three approaches was broadly comparable, differing mainly in throughput and labor cost rather than precision [123]. A mechanically instrumented potato harvester detector finds that simple OpenCV-based thresholding and contour detection performs as the weakest baseline, breaking down entirely under low-light conditions where YOLO-family models continue to work [18]. A rare five-way comparison for real-time conveyor-line potato defect and disease detection reaches a similarly nuanced conclusion. Classical pipelines built around Viola-Jones or Haar-cascade detection or SIFT plus SVM features are outperformed by combinations that pair Otsu thresholding with a CNN or HOG descriptors with a bag-of-visual-words neural network, but only by a modest margin (97% versus up to 95% for the strongest classical variant) [94].

These findings suggest that the largest reported classical-versus-deep-learning gaps in the reviewed literature tend to appear in studies that compare only a single classical baseline, rather than in the few that benchmark several side by side. A well-tuned classical pipeline may match learned models for narrowly defined, geometry-dominated tasks, such as size grading, at a fraction of the computational cost. The choice between the two is better framed as task-dependent than as a simple hierarchy running from classical methods to deep learning.

### 10.6. Limitations of This Review

The limitations of this review fall into two groups, those related to the search and selection of studies and those related to the synthesis. As regards the former, the search covered only two databases, Scopus and Web of Science, and only journal articles. These databases were chosen for their consistent metadata and reproducibility, although relevant studies from other sources may have been missed. Conference papers and proceedings, which form a substantial part of the computer vision and agricultural engineering literature (for example, in IEEE Xplore, ASABE, and CIGR), were not searched, so some methodological work may be missing. For this reason, statements about what is absent from the field, such as the limited use of foundation and zero-/few-shot models, refer to the reviewed corpus and not to the whole literature. The search was run on 17 July 2026, so publications from 2026 are partial, and the counts and shares could change slightly in a later search. Screening was divided between two reviewers, so each record was assessed by one reviewer rather than independently in duplicate. Studies were checked again during data extraction and synthesis to limit the impact of this, although some screening errors cannot be ruled out. No protocol was registered, and no formal risk-of-bias assessment was performed. The search terms were in English, although no language restriction was applied, so studies indexed under terms in other languages may be missing.

As regards the latter, the synthesis is narrative and the counts are descriptive, so the figures reported are not pooled estimates. A meta-analysis was not feasible because the studies differ widely in metrics, techniques, and validation protocols, and it would require much narrower inclusion criteria. This review instead aimed to identify broad, general gaps across the field.

## 11. Future Research Directions

The findings of this review point to several clear gaps and opportunities for future work:

- Multi-modal fusion. RGB, hyperspectral, and depth imaging are well represented, but few studies have combined them, as in a harvester system pairing RGB with depth, or have complemented cameras with NIR point spectra [20,22]. Biospeckle imaging remains largely unexplored in combination with these modalities in root and tuber crops. Fusing RGB defect detectors with spectral models for starch or moisture could allow a single pass to assess both external and internal quality.
- Crop coverage. Potato dominates among the analyzed studies. Other crops (cassava, carrot, oca, water chestnut, porang, and sugar beet) have at most a handful of studies each. Some isolated studies, such as the transformer-based water chestnut detector [38], are methodologically advanced; however, the lack of datasets and research attention limits progress. Applying existing pipelines to two or three underexplored species would be helpful.
- Public datasets and benchmarks. Only five studies have used public datasets. None are dedicated benchmarks for root or tuber crops. Most quantitative comparisons are based on incomparable proprietary data. Shared benchmark datasets with fixed protocols would allow genuine comparison across studies.
- Validation protocols. Most studies used a fixed train/validation/test split. Cross-validation appeared in less than a third of the reviewed studies, and cross-site testing, that is, training on one site and testing on another, remains rare, as in a study that tested on a held-out location [11]. A common protocol based on an independent test set and cross-site validation would make results comparable across studies.
- Deployability and explainability. Few studies target edge deployment, and even fewer evaluate systems in the field. The gap between lab accuracy and real-world efficiency, shown by a system with 98.1% lab accuracy but 61–86% deployed efficiency [48], highlights the need to evaluate systems under deployment conditions. This is most pressing for spectral approaches, which have been validated almost only in the laboratory. Only nine studies (6%) used dedicated interpretability tools, such as SHAP [55] and Grad-CAM [2]; twelve (9%) if feature-importance analyses are also included, although operators would benefit from understanding why a tuber was rejected.
- Foundation models and annotation burden. Foundation models were almost entirely absent from the reviewed studies: only one study incorporates SAM, which is an intermediate-segmentation step within its own pipeline rather than a general-purpose few-shot or zero-shot tool [88]. Approaches that reduce annotation burden, such as few-shot classification or transfer from pretrained models, were rarely tested in the reviewed studies.
- Synthetic data, generalization, and reproducibility. Data augmentation was reported in few studies, and generative models were rarely explored, as in a study that used a GAN [3]. Given the limited size and diversity of most datasets, approaches such as GANs, diffusion models, and simulation could help introduce variations in defects, illumination, and cultivars. However, their effectiveness in improving generalization across acquisition sites remains uncertain. Future studies should evaluate synthetic data on real images and document augmentation procedures, model configurations, and data splits to support reproducibility.
- System-level integration. Most studies evaluate models in isolation. Few studies have assessed the full system from image to action. The harvester with pneumatic ejection [81] and the grading line with reduced deployed efficiency [48] are exceptions. Evaluating the complete pipeline, including throughput and integration with existing systems, would help move advances into practice. One study combined NIR, computer vision, radio-frequency identification (RFID), and blockchain for supply chain traceability [120], a rare example of vision output feeding into a larger system.
- Hardware and system reporting. Future studies should report the hardware used for training and inference separately. For reproducibility, they should also describe processing speed, model size, memory requirements, operating conditions, and the connection between the vision system and the physical control mechanism.
- Artificial intelligence as the next step. Beyond conventional detection and classification, large language models and vision-language models could support quality assessment and decision-making from images or model outputs. Recent decision-oriented services can provide structured predictions, scores, and confidence values from text and images [150]. However, their application to root and tuber crops has not been examined in the reviewed studies, particularly under real-time operating conditions. Comparative studies are needed to assess their accuracy, reliability, latency, and computational requirements against established methods.

## 12. Conclusions

The evidence from 141 reviewed studies shows rapid growth in research on image-based inspection of root and tuber crops, with deep learning becoming dominant. Performance depends on the crop, target attribute, sensor, acquisition conditions, and deployment requirements. Coverage is concentrated on potato (77.3%), whereas cassava and other crops are covered by only a handful of heterogeneous studies, even though the required methods have already been demonstrated on potato. Moreover, most studies were validated under controlled laboratory conditions, and cross-site testing is rare. Reported accuracies may therefore overstate performance outside the conditions in which the systems were developed and evaluated. In addition, differences in data and protocols prevent direct comparison across studies, so this review cannot establish which technique is best for a given task. Where classical and learned methods were compared directly, results were mixed, and a well-tuned classical pipeline can match learned models on simple geometric tasks at a lower cost. Overall, the technical building blocks are largely in place, so progress toward commercial use depends less on new model architectures than on broader crop coverage, shared benchmark datasets, routine cross-site validation, and complete system evaluation.

## Figures and Tables

**Table 1:** Previous reviews related to the non-destructive inspection of root and tuber crops.

Year	Crop(s)	Focus	Imaging Systems	Period and Databases	Ref.
2020	Potato, sweetpotato	Harvested organ: external and internal quality evaluation.	RGB, hyperspectral, NIR, MRI, Raman	Not stated	[29]
2021	Potato, sweetpotato	Harvested organ: quality, defects, and authentication.	Hyperspectral, multispectral	Not stated	[30]
2023	Cassava, yam	Harvested organ: starch, protein, dry matter, amylose, sugars, and texture.	NIR hyperspectral	Not stated	[35]
2023	Potato	Harvested organ: sorting machines and quality classification.	RGB, ultrasound, mechanical	Not stated	[31]
2024	Potato	Plants in the field and harvested organ: production chain, including pests and diseases, plant health, yield and quality prediction, and crop management.	RGB, UAV multispectral and hyperspectral, satellite, stereo depth, Vis/NIR	Web of Science; period not stated	[32]
2024	Potato	Mainly harvested organ and, to a lesser extent, leaves and canopy in the field: compound content, diseases, and stress factors.	Hyperspectral (VNIR, SWIR, fluorescence), multispectral	Web of Science and Scopus; searched June 2023, no year limit	[33]
2025	Potato	Plants in the field and harvested organ: cultivation (weeds, diseases, moisture), harvesting (clods, damage), and storage (defects, sprouting).	RGB; hyperspectral, infrared, and lidar in outlook	Web of Science, Scopus, IEEE Xplore, and Google Scholar; period not stated	[34] *
2027 †	Sweetpotato	Harvested organ: quality of intact roots, sorting prototypes, and validation design.	NIR, hyperspectral, RGB, Raman, MRI	Web of Science Core Collection and X-MOL; January 2020 to August 2026	[36]

Note: * Full text not accessed (payment required). The entry was inferred from the abstract, figures, and reference list available on ResearchGate. † The year is the one indexed in Scopus.

**Table 2:** Number and percentage of studies by crop species.

Crop	*n*	%
Potato (*Solanum tuberosum*)	109 *	77.3
Sweetpotato (*Ipomoea batatas*)	24 *	17.0
Cassava (*Manihot esculenta*)	3	2.1
Carrot (*Daucus carota*)	2	1.4
Oca (*Oxalis tuberosa*)	1	0.7
Water chestnut (*Eleocharis dulcis*)	1	0.7
Porang (*Amorphophallus muelleri*)	1	0.7
Sugar beet (*Beta vulgaris*)	1	0.7

Note: * One study evaluated both potato and sweetpotato and was counted once under each crop.

**Table 3:** Cross-tabulation of studies by inspection task and technique family.

Inspection Task	Image Processing	Traditional ML	Deep Learning	Total Studies
External defect and damage detection	17	16	25	43
Sorting and grading (size, shape, weight, and quality)	20	13	8	27
Disease and pest detection	6	6	13	17
Field phenotyping, counting, and yield estimation	7	2	5	12
Non-destructive internal-quality estimation	4	10	4	13
Foreign object/impurity detection	1	4	10	13
Freshness and storage quality estimation	3	5	3	8
Sprouting and greening monitoring	1	2	2	5
Variety/cultivar identification	2	2	1	3

Note: Technique-family columns are not mutually exclusive.

**Table 4:** Summary of the sensing modalities reported in the included studies.

Modality	*n* (%)	Typical Device(s)	References
RGB	112 (79%)	Industrial area/line-scan cameras; DSLR/mirrorless; smartphone	[18,21,56,57,58]
Hyperspectral	27 (19%)	Pushbroom VIS-NIR/SWIR line-scan cameras (e.g., SOC710-VP, Pika XC2, and SPECIM FX17)	[13,19,22,59,60,61,62,63,64]
Near-infrared spectroscopy (non-imaging, complementary to cameras)	5 (4%)	Point/micro-NIR spectrometers used alongside RGB or hyperspectral cameras	[20,21,22,63,65]
3D/Depth	17 (12%)	Intel RealSense L515 (ToF); multi-camera SfM rigs	[10,14,51,66,67,68,69]
Multispectral	4 (3%)	Discrete-band interactance/reflectance cameras and snapshot mosaic sensors	[70,71,72]
Thermal (LWIR)	6 (4%)	Infrared thermographic camera	[4,15,73,74,75,76]
X-ray/CT	1 (0.7%)	Computed tomography (CT) scanner (paired with MRI/NMR in the same study)	[17]
Laser backscattering/biospeckle	4 (2.8%)	Coherent laser illumination + line scan or speckle pattern camera	[7,16,69,77]

Note: Total *n* = 141; category counts are summed to more than 141 and percentages to more than 100% because a single study commonly uses more than one acquisition modality (e.g., both a spectral and an RGB camera). For the full per-study breakdown, see Supplementary Table S1. Abbreviations: NIR: near-infrared; 3D: three-dimensional; LWIR: long-wave infrared; CT: computed tomography; MRI: magnetic resonance imaging; NMR: nuclear magnetic resonance; DSLR: Digital Single-Lens Reflex.

**Table 5:** Processing hardware and deployment patterns.

Hardware Layer	General Pattern	Approximate Evidence	Relevance for Deployment	Example Devices Used
Training and offline processing	GPU-based processing was common in deep-learning studies. NVIDIA was the most frequently identified manufacturer.	Nearly half of the studies reported a GPU or another accelerator.	Reported performance often reflects hardware used for development rather than operation in the field.	RTX 4060 [46], RTX 3090 [5], RTX 4090 [81,112], NVIDIA V100 [113], Tesla P100 through Google Colab [86,116]
CPU and memory	CPU and RAM specifications were reported less consistently than GPU information.	Around one third of the studies reported CPU or memory details.	Missing specifications limit the comparison and reproduction of computational requirements.	Intel Core i5 and i7 systems [3,41,80], Intel Xeon with RTX A6000 [83], AMD EPYC with RTX 3080 [111]
Inference and deployment	Only a small group separated training hardware from inference hardware.	Fewer than ten studies described both platforms separately.	The hardware used for real-time operation may impose additional limitations on speed and memory.	Jetson Orin Nano [5,46], Jetson AGX Orin [81], Jetson Xavier NX [113], industrial computer with RTX 3070 [111]
Edge and embedded processing	Jetson, Raspberry Pi, and microcontrollers were used for local processing or system control.	18 studies (13%) included edge or embedded hardware.	These platforms can support local operation but require evaluation under actual power, memory, and timing constraints.	Jetson-based systems [56,81,93], Raspberry Pi [45,83,114], Arduino-based control [48,51,88]
PLC and industrial control	PLCs were mainly associated with conveyors, pneumatic systems, motors, and rejection mechanisms.	Approximately ten studies provided concrete evidence of this integration.	System performance depends on the complete sequence from image acquisition to physical actuation.	IPC and PLC [92,107], pneumatic or stepper actuation [47,112], Mitsubishi FX3U PLC [73], PLCnext with Raspberry Pi [115]
Complete system operation	Most studies evaluated the model or imaging system separately from the physical sorting process.	Only a small number reported the complete vision-to-action pipeline.	Accuracy alone does not describe throughput, synchronization, or mechanical performance.	Conveyor sorting [47,81,111], automated grading with mechanical ejection [48], industrial sorting systems [73,92,107]

Note: Hardware categories could overlap because a study could use one platform for training, another for inference, and additional devices for control or actuation.

**Table 6:** Classical digital image processing techniques identified in the reviewed studies, their typical purpose, and representative studies.

Technique	*n* (%)	Typical Purpose	References
Generic/other thresholding	48 (34%)	Foreground–background separation before feature extraction or a learned model	[48,98,117,118,119,120]
Grayscale conversion	36 (26%)	Discarding color before thresholding or edge detection	[121,122]
Morphological operations (e.g., erosion, dilation, opening, and closing)	34 (24%)	Mask clean-up: removal of artifacts, separation of touching tubers, and smoothing of boundaries	[48,98,117,123,124]
Background subtraction/removal	22 (16%)	Isolation of the tuber from a controlled imaging background	[3,89]
HSV color-space transformation	17 (12%)	Illumination-robust color/hue-based segmentation or description	[28,37]
GLCM texture descriptors	14 (10%)	Quantification of surface roughness/texture as classifier input	[8,23,99]
Otsu thresholding	12 (9%)	Automatic data-driven threshold selection	[117,125]
Canny/edge detection	11 (8%)	Delineation of the boundaries and cracks/defects	[109,110,121,126]
L*a*b* (CIELAB) color-space transformation	5 (4%)	Perceptually uniform quantification of subtle color differences	[16,72]
Fractal-dimension descriptors	2 (1.4%)	Quantification of the surface irregularity	[37,57]
Fourier shape descriptors	2 (1.4%)	Quantification of boundary irregularity/deformity for shape-based grading	[127,128]
Watershed segmentation	1 (0.7%)	Separation of the touching or overlapping regions	[123]

Note: Total *n* = 141; category counts overlap because a single study commonly applies more than one classical technique. For the full per-study breakdown, see Supplementary Table S2. Abbreviations: HSV: Hue-Saturation-Value.

**Table 7:** Representative deep learning architectures reported in the included studies, by crop, inspection task, and best reported performance.

Study	Crop	Task	Architecture/Backbone	Best Reported Performance
[46]	Potato	Object detection (foreign objects/defects, 4 classes)	MCDE-YOLOv11 (directional attention, multiscale conv.)	mAP@0.5 97.7%
[41]	Potato	External defect detection (7 classes)	HCRP-YOLO (pruned YOLOv8n, HGNetv2 backbone)	mAP 95.3%
[93]	Potato	Object detection/material classification (field)	WTDE-DETR (RT-DETR + wavelet attention)	mAP@0.5 95.0%
[92]	Potato	Foreign-object detection (tuber vs. soil/stone)	CenterNet variant (anchor-free and ACmix attention)	97.5% accuracy
[82]	Sweetpotato	Semantic + instance segmentation (skinning %)	DeepLabV3+ (ResNet50) + Mask R-CNN	99.98% instance grading accuracy
[75]	Potato	Disease and pest detection	ResNet18 (vs. VGG16/19, ResNet50/101, mod. AlexNet)	62.9% accuracy
[70]	Sweetpotato	Root-knot nematode gall detection (galled vs. healthy, binary)	Transfer learning: ResNet101/50/18, MobileNetV2, InceptionV3, and NASNetMobile	97.5% accuracy
[131]	Sweetpotato	External defect severity grading (4 classes)	SP-YOLOv11 (YOLOv11s + adaptive attention, multiscale fusion)	mAP@0.5 98.5%
[54]	Sweetpotato	In-field phenotyping (root instance counting/yield)	Mask R-CNN (instance segmentation)	AP@0.5 97.5%; R^2^ 0.80 (per-plot count)
[86,116]	Sweetpotato	Internal quality (dry matter/soluble solids via RGB-to-hyperspectral reconstruction)	HSCNN-D/HRNET/MST++ + PLSR + SHAP	R^2^p 0.88, RPD = 2.93 (dry matter, [86]); R^2^p 0.70, RPD = 1.82 (soluble solids, [116])
[132]	Potato	Multi-task disease detection + quality grading (joint model)	Multi-task CNN + YOLO11m ROI extraction benchmarked against Ridge/Lasso/SVR/Random Forest/XGBoost	Accuracy, 90.8%; F1, 0.931 (multi-task model); R^2^ 0.72 (SVR on ConvNeXt-Base features)
[133]	Potato	Discrimination between freeze damage, mechanical damage, and bruising (hyperspectral)	Boosting ensemble (XGBoost, CatBoost, and LightGBM) + stacking on hyperspectral signatures	98.9% accuracy
[76]	Potato	External + internal quality assessment (thermography + machine vision fusion)	Active thermal imaging + digital BOF descriptors + fuzzy ARTMAP neural network (vs. backpropagation/perceptron)	100% (best configuration)

Note: This table lists a representative subset of included studies, chosen to illustrate the leading architecture for each crop and inspection task; see Supplementary Table S3 for the full per-study breakdown. Abbreviations: mAP: mean average precision; AP: average precision; R^2^: coefficient of determination; RPD: ratio of performance to deviation; F1: F1 score; ROI: region of interest; BOF: Boundary Object Function; PLSR: Partial Least Squares Regression; SHAP: SHapley Additive exPlanations.

**Table 8:** Representative studies by inspection task, indicating crop(s), and representative technique(s).

Inspection Task	Crop(s)	Representative Technique(s)	References
Sorting/grading (size, shape, and weight)	Potato, sweetpotato, and carrot	Linear SVM; YOLOv8.1 + SAM segmentation; multi-view RGB-D; Mask R-CNN instance segmentation; cross-location YOLO benchmarking.	[5,7,11,48,88,98,106,134,135]
External defect, damage, and skin disorder	Potato, sweetpotato	YOLO-based detection (HCRP-YOLO, SP-YOLOv11, and YOLOv3/v5s variants); GLCM texture classification; CNN classification; weakly supervised localization.	[18,40,41,43,71,73,97,99,102,111,112,113,115,131,136,137,138,139]
Disease and pest detection	Potato, sweetpotato	Color/texture classifiers; CBAM-YOLOv8; CNN classifiers; hyperspectral (Zebra Chip, frost/disease discrimination).	[27,44,85,94,108,125,140,141]
Sprouting, greening, and physiological disorders	Potato	Hyperspectral ΔE thresholding; MC-CNN; chlorophyll/TGA regression.	[90,103,114,142]
Non-destructive assessment of the internal quality	Potato, sweetpotato, and cassava	PLSR/CARS-SVR; blackheart classifiers; spectral regression; RGB-to-hyperspectral reconstruction; thermal-property estimation.	[12,13,20,74,87,104]
Variety/cultivar identification	Potato	MLP; SqueezeNet; CNN classification.	[24,100,128]
Freshness, ripeness, and storage quality	Potato, cassava	Commodity + freshness classifier; binary freshness/age regression; kNN/classical ensemble.	[4,6,143,144,145]
In-field phenotyping, counting, and yield analysis	Potato, sweetpotato	Harvester-mounted detection; RGB-D + stereo photometry; hyperspectral regression; Mask R-CNN instance counting; high-throughput bin-scanning phenotyping platforms.	[52,53,54,81,101,105]
Integration of real-time/robotic/industrial sorting-line	Potato and water chestnut	Color/shape and color/texture classification; ANN; YOLOv5; RT-DETR; YOLOv12; laser backscattering.	[1,38,47,56,77,95,146,147]

Note: This table lists representative, non-exhaustive studies for each inspection task, not a full per-study breakdown; see Supplementary Table S4 for the complete listing. Abbreviations: MC-CNN: multiple channels convolutional neural network; PLSR: Partial Least Squares Regression; CARS: Competitive Adaptive Reweighted Sampling; TGA: Total Glycoalkaloids.

**Table 9:** Public or repository-hosted datasets identified in the literature review.

Dataset Name/Source	Crop	Modality	Reported Size	Used by
Potato-Disease/PotatoCare/PotatoGAN (combined)	Potato	RGB	451/3891/192 images	[148]
Fruits 360 (filtered subset)	Potato	RGB	2316 images (filtered)	[6]
Multi-repository compilation (Kaggle, Google, Purdue, and others)	Sweetpotato	RGB	25,988 images	[25]
Kaggle cassava dataset + own field images	Cassava	RGB	Not specified (public portion) + field validation set	[45]
Kaggle vegetable-disease dataset (potato subset)	Potato	RGB	380 of 1030 images	[149]

## Data Availability

Files used in the bibliometric analysis, including the literature search exports, are available in the Zenodo repository: https://doi.org/10.5281/zenodo.22805392.

## Supplementary Materials

The following supporting information can be downloaded at https://www.mdpi.com/article/10.3390/foods15213903/s1, Table S1: Full per-study breakdown of sensing modality; Table S2: Full per-study breakdown of classical image-processing techniques; Table S3: Full per-study breakdown of machine-learning and deep-learning architecture families; Table S4: Full per-study inspection-task listing; Table S5: Per-study self-reported limitations and interpretability/explainability technique breakdown.

## Abbreviations

The following abbreviations are used in this manuscript:

ANN	Artificial Neural Network
AP	Average Precision
ATO	Automatic Training Once
AUC	Area Under the Receiver Operating Characteristic Curve
BOF	Boundary Object Function
CARS	Competitive Adaptive Reweighted Sampling
CBAM	Convolutional Block Attention Module
CIELAB	CIE L*a*b* Color Space
CNN	Convolutional Neural Network
CT	Computed Tomography
CV	Computer Vision
DL	Deep Learning
DSLR	Digital Single-Lens Reflex
ELM	Extreme Learning Machine
F1	F1 score
FPGA	Field-Programmable Gate Array
FPS	Frames Per Second
GAN	Generative Adversarial Network
GLCM	Gray-Level Co-occurrence Matrix
Grad-CAM	Gradient-weighted Class Activation Mapping
HOG	Histogram of Oriented Gradients
HSV	Hue-Saturation-Value
IoU	Intersection over Union
IP	Image Processing
kNN	k-Nearest Neighbors
LSTM	Long Short-Term Memory
LWIR	Long-Wave Infrared
mAP	mean Average Precision
MC-CNN	Multiple Channels Convolutional Neural Network
ML	Machine Learning
MLP	Multilayer Perceptron
MRI	Magnetic Resonance Imaging
NIR	Near-Infrared
NMR	Nuclear Magnetic Resonance
PCA	Principal Component Analysis
PLS	Partial Least Squares
PLS-DA	Partial Least Squares Discriminant Analysis
PLSR	Partial Least Squares Regression
R^2^	Coefficient of Determination
R-CNN	Region-based Convolutional Neural Network
RF	Random Forest
R-FCN	Region-based Fully Convolutional Network
RFID	Radio-Frequency Identification
RGB-D	Red-Green-Blue-Depth
RMSE	Root Mean Square Error
ROI	Region of Interest
RPD	Ratio of Performance to Deviation
RT-DETR	Real-Time Detection Transformer
SAM	Segment Anything Model
SHAP	SHapley Additive exPlanations
SIFT	Scale-Invariant Feature Transform
SIMCA	Soft Independent Modeling of Class Analogies
SMOTE	Synthetic Minority Oversampling Technique
SSD	Single Shot Detector
SVM	Support Vector Machine
SVR	Support Vector Regression
TGA	Total Glycoalkaloids
YOLO	You Only Look Once
